# Review of Optical Humidity Sensors

**DOI:** 10.3390/s21238049

**Published:** 2021-12-01

**Authors:** Xing Rao, Lin Zhao, Lukui Xu, Yuhang Wang, Kuan Liu, Ying Wang, George Y. Chen, Tongyu Liu, Yiping Wang

**Affiliations:** 1 Key Laboratory of Optoelectronic Devices and Systems of Ministry of Education/GuangDong Province, College of Physics and Optoelectronic Engineering, Shenzhen University, Shenzhen 518060, China; 2060453012@email.szu.edu.cn (X.R.); 2100453032@email.szu.edu.cn (L.X.); 2100453052@email.szu.edu.cn (Y.W.); 2110456067@email.szu.edu.cn (K.L.); yingwang@szu.edu.cn (Y.W.); ypwang@szu.edu.cn (Y.W.); 2 Shenzhen Key Laboratory of Photonic Devices and Sensing Systems for Internet of Things, Guangdong and Hong Kong Joint Research Centre for Optical Fibre Sensors, Shenzhen University, Shenzhen 518060, China; 3 Laser Institute, Qilu University of Technology (Shandong Academy of Sciences), Jinan 250353, China; linzhao1225@126.com (L.Z.); tongyu.liu@vip.iss-ms.com (T.L.)

**Keywords:** humidity, RH, moisture, hygrometer, optical fiber, review

## Abstract

Optical humidity sensors have evolved through decades of research and development, constantly adapting to new demands and challenges. The continuous growth is supported by the emergence of a variety of optical fibers and functional materials, in addition to the adaptation of different sensing mechanisms and optical techniques. This review attempts to cover the majority of optical humidity sensors reported to date, highlight trends in design and performance, and discuss the challenges of different applications.

## 1. Introduction

The measurement of relative humidity (RH) is an important part of heating, ventilation, air conditioning, and refrigeration (HVACR), which provides quality control in numerous aspects of daily life. The full range of applications include: (a) manufacturing (e.g., paints/coatings, construction timber, greenhouses); (b) refrigeration (e.g., industrial, residential homes); (c) packaging (e.g., food, dry commodities); (d) transportation (e.g., cargo bay, in-cabin climate control, under the hood); (e) building conditioning (residential homes, museums, heritage buildings); and (f) healthcare (e.g., incubators, patient monitoring, respiratory, ventilators); (g) agriculture/forestry (e.g., plantations, grain storage, water regulation); (h) touchless control systems (e.g., building access, luxury vehicles); (i) weather stations (e.g., weather forecasts, climate studies, bushfire prevention); hence, an accurate and reliable means to monitor RH supports the development and operation of numerous industries, potentially connected together under the internet of things (IoT).

Owing to different environments and requirements, a wide variety of humidity-sensing technologies (e.g., humidity sensors or hygrometers) have been researched and developed in the literature, including optical/photonic/optoelectronic [1,2,3], quartz crystal microbalance (QCM) [4,5], capacitive [6] and resistive [7]. Apart from the known advantages of immunity to electromagnetic interference (EMI) and electrical inertness, optical-based humidity sensors are typically more sensitive and offer a broader range of capabilities tailored for different applications (e.g., colorimetric, point, distributed) [8] compared to their traditional counterparts. However, they are often bulkier and more expensive [9] due to the conversion between light and electricity. Optical humidity sensors can rely on a multitude of parameters, such as transmitted power, wavelength, frequency, and phase. The former is the simplest approach and is employed for low-end humidity measurement applications, where high accuracy is not a stringent requirement.

**Figure 1:**
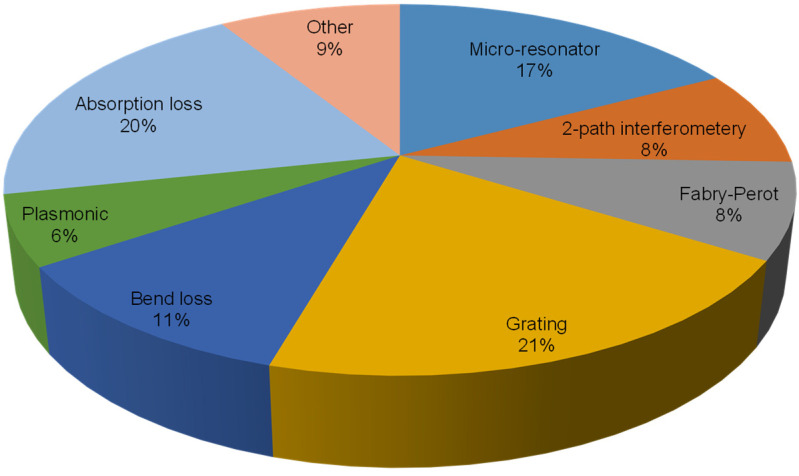
Types of sensing mechanisms for point humidity sensors and their usage percentage.

**Figure 2:**
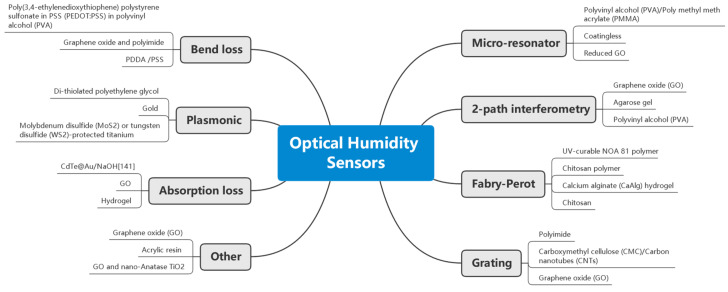
Example functional materials for each type of sensing mechanism.

Optical humidity sensors include colorimetric indicators, control systems, point sensors, and distributed sensors. Among point sensors, the most common types involve gratings and absorption loss (Figure 1), with each accounting for approximately a fifth of the total number of publications. Recent new sensors often emphasize developing new functional materials for novelty and performance improvement (Figure 2) in terms of sensitivity, response time, and specificity. It can be seen that a great variety of materials have been explored for various sensing mechanisms. In this review, a comprehensive summary of the reported works is presented in chronological order (by year) for each class. Comparisons are made between humidity sensors based on specific attributes. The commonly used functional materials are presented. The existing technical challenges are discussed, and an outlook is unveiled for the future.

## 2. Basic Principles

Relative humidity (RH) indicates the percentage of water vapor in a water-air mixture relative to the saturation level (i.e., onset of water droplets formation) at a given temperature. Hence, at a lower temperature, 100%RH can be achieved with fewer water molecules. This effect can be observed with the common phenomenon of condensation on cooler surfaces. For simple testing of a humidity sensor, the amount of water molecules is varied instead of the temperature. This is because changing the temperature could lead to unwanted temperature-related effects, including thermo-optic, stress-optic, and thermal expansion.

**Figure 3:**
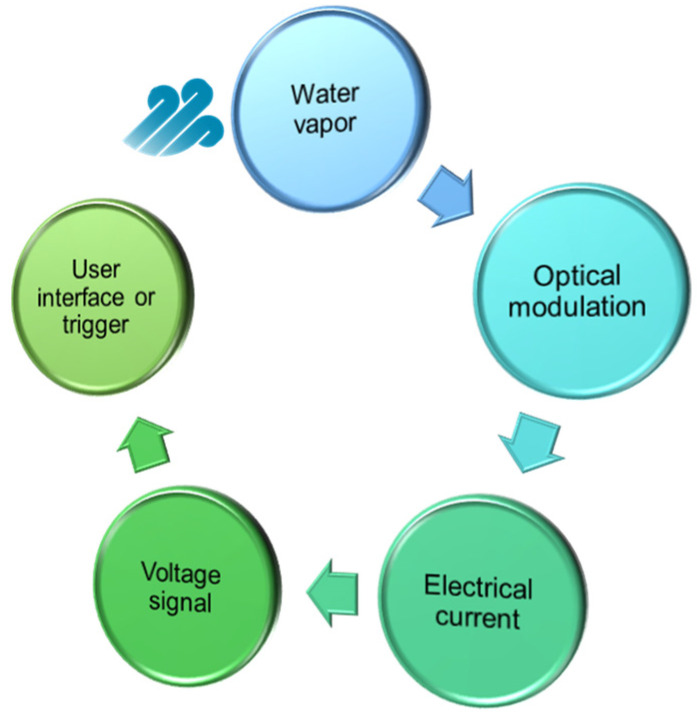
Flow diagram of the operation of a typical optical humidity sensor.

A humidity sensor is defined as a device that provides a measure of the RH and either presents the information directly to the user or serves as an actuator to drive the next stage in a larger system, as illustrated in Figure 3.

## 3. Literature and the State-of-the-Art

### 3.1. Colorimetric Indicators

Low-cost functional strips providing a means to assess the approximate level of RH by eye are useful for electronics, greenhouses, and patient monitoring. Typically, accuracy and response time are not prioritized, as the checks are performed manually. On the contrary, color uniformity and dynamic range are considered important for the end-users. Two or more colorimetric strips of different color maps can be used in conjunction to improve the accuracy of readings.

The demand for a readily accessible humidity indicator that is vibration resistant likely peaked during World War II, when people became concerned about the poor condition of weapons and ammunition. High levels of humidity combined with poor packaging methods led to corrosion and moisture damage. In the beginning, the concept for the first color-change humidity indicator involved a simple go/no-go method of measuring humidity. In the late 1940s, RHs in the range of 30%RH–35%RH were considered important because it is the onset for corrosion [10]. For the next few decades, industrial and military applications for colorimetric humidity indicators dominated the market. In the mid-1980s, manufacturers of semiconductors were concerned with the delamination of solder surface-mounting of semiconductors when they had been exposed to a high level of moisture within the package. As a result, an industry-wide standard for packaging semiconductors was established, with the inclusion of a humidity indicator to inform the manufacturer that the device has been exposed to excessive moisture. In 1999, J-STD-033 was adopted with the availability of 5%RH, 10%RH, and 15%RH color-change indicator cards.

**Figure 4:**
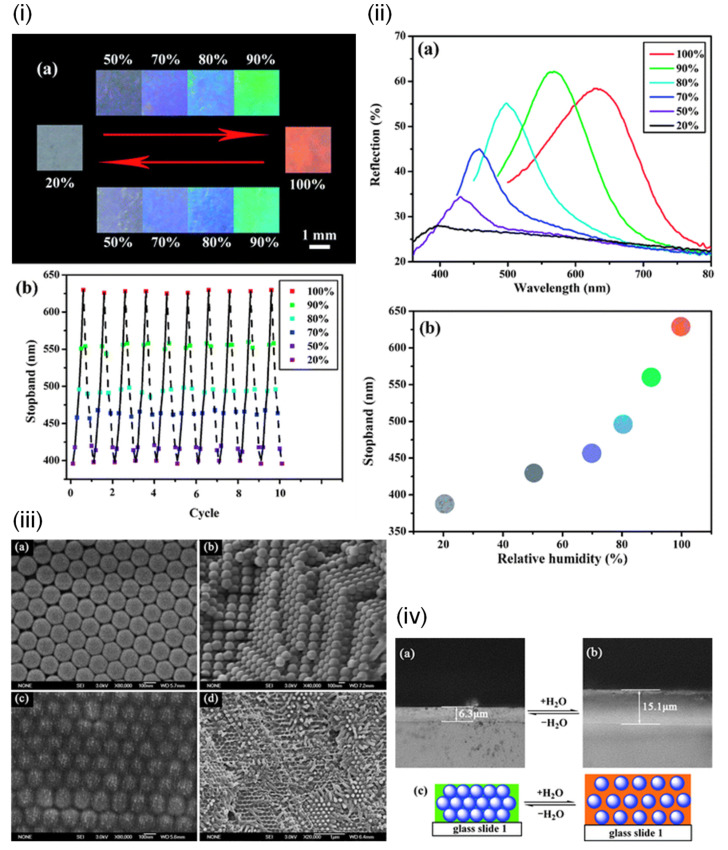
Colorimetric indicator. (**i**) Reversible changes of (**a**) color and (**b**) stopband with changing RH. (**ii**) (**a**) UV-vis spectra of the films under different RH. (**b**) Relationship between the stopband and RH. (**iii**) Scanning electron microscope (SEM) images of the fabricated hydrogel. P(St–MMA–AA) PC seen from the (**a**) top and (**b**) cross-view. PAAm–P(St–MMA–AA) observed from the (**c**) top and (**d**) cross-view. (**iv**) Microscope images of the hydrogel in its (**a**) dry state and (**b**) wet state. (**c**) Illustration of the structure change of hydrogel before and after fully wetted in water. Adapted with permission from ref. [11]. Copyright 2008 *Royal Society of Chemistry*.

Among the earliest humidity-sensitive colorimetric indicators was one reported by Tian et al. in 2008 [11] (Figure 4). The team demonstrated a colorimetric photonic crystal hydrogel that responds to changes in RH with up to a 240 nm shift in wavelength, which can cycle through transparent to violet, blue, cyan, green, and red under different RH levels. The coating on a glass slide was fabricated by infiltrating acrylamide (AAm) solution into a poly(styrene-methyl methacrylate-acrylic acid) P(St–MMA–AA) photonic crystal template followed by photo-polymerization. The optical response is due to the Bragg effect. Zhanhua Wang et al. [12] created bioinspired humidity-sensitive organic/inorganic hybrid photonic crystals. These one-dimensional crystals were able to tune across 147 nm in the visible spectrum with changing RH. The response time was measured to be 150 s. The material can be fabricated by alternating thin films of titania and poly(2-hydroxyethyl methacrylate-co-glycidyl methacrylate) (PHEMA-co-PGMA) via spin coating. The optical response is also due to the Bragg stack of layers.

Matthew M. Hawkeye et al. [13] developed a mesoporous TiO_2_-based photonic crystal material. A 40 nm spectral shift was demonstrated by varying the RH, and a 1%RH was claimed to be visually distinguishable. The colorimetric indicator was fabricated on silica glass using glancing angle deposition (GLAD). The color mechanism is also based on Bragg resonance. Hong Chi et al. [14] used a different approach to the Bragg effect and fabricated a colorimetric indicator using 2D graphene oxide (GO) nanosheets. A 119 nm spectral shift with a 250 ms response time was achieved. The coating on a silicon substrate was performed using dip coating. The color mechanism, in this case, is simply absorption, swelling, and thin-film interference.

Andrew Mills et al. [15] reported a heat-activated colorimetric indicator based on methylene blue encapsulated in hydroxypropyl cellulose (HPC). Upon heat treatment at 370 °C for 4 s, the indicator responds to an environment above 70%RH, with up to 135 nm spectral shift. However, within seconds, the color reverts back to the default inactive state. Nattinee Bumbudsanpharoke et al. [16] applied photonic cellulose nanocrystals for measuring RH, which operates under a polarized optical microscope, with up to 135 nm spectral shift and a response time of 240 min. Katerina Lazarova et al. [17] spin-coated poly(vinyl alcohol-co-vinyl acetal)-based thin films and performed reflectance measurements at different RH in the range of 5%RH–95%RH.

### 3.2. Touchless Control Systems

Conventional touchless control inputs are either based on voice (i.e., long range), motion (i.e., medium range), or capacitance (i.e., short range). Such controls enable the non-contact operation of gadgets, machinery, or infrastructure, which has the benefits of convenience and hygiene. While these technologies work fine most of the time, false inputs are a possibility. With voice input, background noise can sometimes interfere and become misinterpreted as valid commands. Motion commands can be erroneously entered if the foreground contains multiple moving objects or people. Even with capacitance-based controls, static charge and liquid droplets can hinder the ability of the system to distinguish user inputs from random perturbations. On the other hand, humidity-based control can be more reliable, though possibly limited in variety. The humidity signal can be generated by human breath or finger proximity, and changes are much faster than that of the ambient environment, which lends itself to easy recognition.

In 2011, Jinish Mathew et al. [18] demonstrated an optical switch based on high bend-loss fiber coated with polyethylene oxide (PEO), which relies on humidity thresholds to switch between different states. The on/off switch operates in the region of 80%RH–95%RH with a response time of 785 ms. The coating was fabricated by dip coating the fiber in the solution. The switch operates by using the transfer of strain from the coating to the fiber, inducing a bend loss. Though not strictly an optical sensor, Jun Feng et al. [19] presented work on a touchless positioning interface using ultrathin VS_2_, nanosheets that respond to humidity with changes in potentially spatially resolvable color (i.e., require greater thickness) and electrical resistance. A variation of 0–320 kΩ was observed for the full RH range. The response time is 30 s, which limits the applications to those with slow-moving inputs. The VS_2_ nanosheet coating was first prepared in solution with a modified liquid-exfoliation method and then transferred to a polyethylene terephthalate (PET) substrate with a higher surface energy for suitable adhesion force. The operating principle involves moisture interaction with the interval structures of the nanosheet, as well as the dynamics of exposed metal atoms, which influences its electrical resistance.

**Figure 5:**
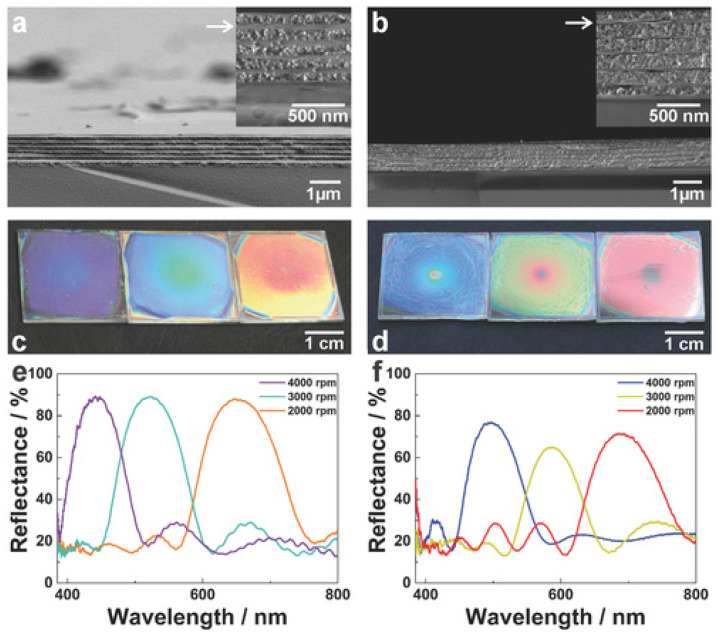
Touchless control system. (**a**) SEM cross-section images of the SiO_2_/H_3_Sb_3_P_2_O_14_ film fabricated by 4000 rpm spin-coating speed. (**b**) SEM cross-section images of the TiO_2_/H_3_Sb_3_P_2_O_14_ film fabricated at the same spin-coating speed. (**c**) Photographs of the SiO2/H3Sb3P2O14 Bragg stacks produced at different spin-coating speeds, from left to right: 2000, 3000, and 4000 rpm, respectively. (**d**) Photographs of the TiO_2_/H_3_Sb_3_P_2_O_14_ BSs produced at the same spin-coating speeds. (**e**) Spectra of the SiO_2_/H_3_Sb_3_P_2_O_14_ films shown in (**c**). (**f**) Spectra of the TiO_2_/H_3_Sb_3_P_2_O_14_ films shown in (**d**). Reprinted with permission from ref. [20]. Copyright 2015 *Wiley*.

Katalin Szendrei et al. [20] (Figure 5) also developed a colorimetric coating for touchless control. Using a different approach based on Bragg stacking of 1D photonic crystals, which also enable transparency for potential application as smart windows. The maximum wavelength shift is 00 nm across the visible region, under a response time of 2 s. The layered phosphatoantimonate K_3_Sb_3_P_2_O_14_ and the proton exchanged phosphatoantimonic acid H_3_Sb_3_P_2_O_14_ were synthesized by a conventional solid-state reaction followed by ion exchange with HNO3. Dropcasting the resulting suspension onto a quartz substrate leads to randomly overlapping, c-oriented nanosheets. The coating absorbs water vapor and swells in thickness, resulting in a change in resonance condition and thus a shift in Bragg wavelength (i.e., perceived color). Li Yu et al. [21] developed a polyelectrolyte coating for touchless control with poly(diallyldimethylammonium) (PDDA)/poly(styrenesulfonate) (PSS) layers, which showed vivid color changes in response to varying RH. The shift in the spectrum can be as high as 305 nm, which spans the entire visible regime. The response time is only 35 ms. The PDDA/PSS was attached to a silicon substrate via dip coating and layer-by-layer assembly. Although multiple layers are involved, the coating behaves as an optically homogeneous layer. The response mechanism is the absorption of water vapor, swelling, change in coating thickness, and refractive index.

### 3.3. Point Sensors

Point humidity sensors provide a single spatial reading of RH and are the most common type due to their simplicity, high sensitivity, and relatively low cost. Some humidity sensors feature a single-ended detection method, where the sensor head is a probe, which is useful for measuring confined environments. Point humidity sensors come in a huge variety of forms and sensing mechanisms, which makes comparing the sensitivities meaningless when the units are different. Instead, the limit of detection (LOD) is compared, which also takes into account the electro-optic devices used for signal conversion.

The earliest optical-based point humidity sensors were reported in the 1980s. Hermann Posch et al. [22] showed the use of perylenedibutyrate dye on optical fiber for measuring RH. A light-emitting diode was used to excite the dye, which lowered the cost. A LOD of 1%RH was demonstrated between 0%RH and 70%RH, with a sensitivity of 1.86%/%RH or 0.0009 dB/%RH between 0%RH and 70%RH. The response time is 5 min. The dynamic range is 0%RH–100%RH. The perylenedibutyrate was adsorbed on a filter paper (i.e., silica gel was inferior) and probe light excited fluorescence on the sheet before being collected with a second fiber. The sensor operates by quenching fluorescence by water vapor, though oxygen interferes in the process. W. Lukosz et al. [23] applied interferometry to an uncoated integrated surface waveguide to measure small changes in RH. They achieved a LOD of 0.1%RH with a response time of 100 ms. The waveguide simply absorbs moisture to increase its effective refractive index, and the corresponding phase shift is detected by the differential detection of the individual polarization states.

Yoshihiko Sadaoka et al. [24] demonstrated a humidity sensor exploiting absorption of probe light in a composite thin-film of Nafion dye, using an optical fiber to deliver and collect light. The response time is approximately 1 min. Composite films were made from a mixture of hydrolyzed Nafion and dye. Thin films of hydrolyzed Nafion were prepared on alumina substrates by dip or spin coating. The sensor works by correlating the absorption of specific wavelength(s) with known RH levels. Owing to the different probe-light absorption rates for different wavelengths, the film changes color with varying RH. Masanori Ando et al. [25] created a Co_3_0_4_-based thin-film colorimetric indicator that can be interrogated by electro-optics. The response time is 1 min, and the sensitivity is 4.9 × 10^−5^ dB/%RH (400 nm wavelength) between 10%RH and 90%RH. Co_3_0_4_ films were fabricated by pyrolysis of a thin layer of cobalt 2-ethylhexanoate, which was then spin-coated on a glass substrate from a mixed solution of toluene and 1-butanol. The film reacts to different RH due to the water-induced absorption of the probe light.

**Figure 6:**
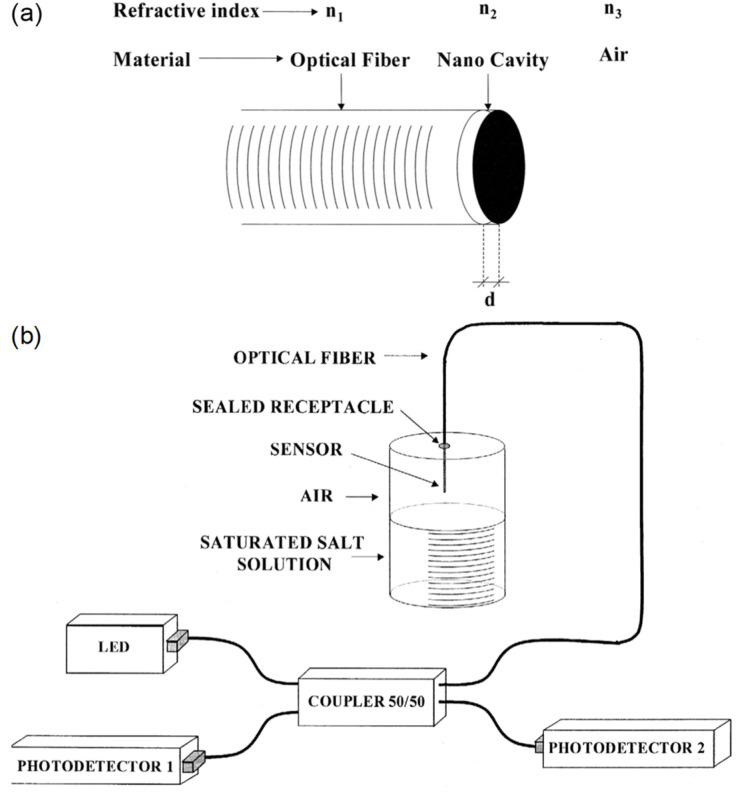
Point sensor. (**a**) Thin-film interferometric cavity. (**b**) Experimental setup. Adapted with permission from ref. [26]. Copyright 1999 *Elsevier*.

A fiber-optic humidity sensor was developed by Francisco J. Arregui et al. [26] (Figure 6), which consists of a fiber-tip Fabry-Perot interferometer (FPI) cavity made up of self-assembled polyelectrolyte of PDDA/PSS. The humidity probe exhibited a response time of 1.5 s. The sensitivity is 0.05 dB/%RH between 11%RH and 100%RH. The ionic self-assembly monolayer (ISAM) technique was used to construct the nano-scale cavity, which involves alternate dip coating. The working principle exploits the hydrophilic nature of the monolayers that absorbs water vapor and swell, resulting in a change in the optical path length (i.e., thickness, refractive index) and end-reflection. The latter is the nominal effect, and thus changes in the reflected power are used to provide a readout of the RH. Cándido Bariáin et al. [27] demonstrated a tapered fiber coated with agarose gel for a humidity sensor. The response time is 5 s, and the sensitivity is 0.14 dB/%RH between 30%RH and 80%RH. The original optical fiber was tapered using an electric splicer. Agarose gel can be prepared by dissolving its powder form in heated water before being deposited on the fiber. The sensing principle involves laser absorption in water.

Francisco Arregui et al. [28] used an alternative functional material for humidity sensing with a fiber tip, using poly R-478 and PDDA. The response time is 2 s. The sensitivity is 0.12 dB/%RH between 11%RH and 85%RH. The fabrication process is again ISAM, and the sensors operate by water absorption of the probe light. Pascal Kronenberg et al. [29] presented an intrinsic humidity sensor using polyimide-coated fiber Bragg gratings (FPGs). The sensitivity is 2.21 × 10^−6^ a.u./%RH between 10%RH and 90%RH. The polyimide coating is hydrophilic and thus swells in humid environments as the water molecules migrate into the polymer. The overlap between the evanescent waves of light and the water molecules within the polymer gives rise to attenuation of light, providing an indication of the RH level.

A plastic optical fiber (POF)-based humidity sensor was developed by Rajeev Jindal et al. [30], with the addition of a thin film of polyvinyl alcohol (PVA) and CoCl_2_. The sensitivity with respect to RH is very irregular between 3%RH and 90%RH, while the response time is in the order of seconds. Prior to coating, the optical fiber was stripped of its cladding and annealed to conform to a U-bend probe. The sensing section was then functionalized with the aqueous solution of PVA and CoCl_2_ before being left to dry. The coating swells with increasing RH and bends the fiber, thus attenuating the light through bend-induced optical leakage. Ainhoa Gaston et al. [31] reported a humidity sensor, among temperature and pH sensors, using optical fiber and PVA coating. The response time is 1 min, and the sensitivity is 0.5 dB/%RH between 70%RH and 90%RH. The optical fiber was side-polished before PVA was applied to the surface by dip coating. The operating mechanism comprises water-induced swelling of the PVA film, which causes an interplay of optical confinement and water-induced absorption of the probe light. As a result, the relationship between transmitted power and RH is not monotonic, with a reversal in trend around 67%RH.

**Figure 7:**
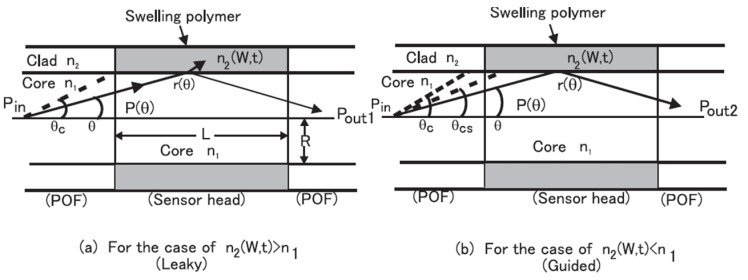
Point sensor. A simulation model of the POF-based humidity sensor involving ray optics. (**a**) For the case of leaky rays (**b**) For the case of guided rays. Reprinted with permission from ref. [32]. Copyright 2003 *IOPscience*.

Shinzo Muto et al. [32] (Figure 7) exploited the combination of POF, hydroxyethylcellulose (HEC), and polyvinylidenefluoride (PVDF) for humidity sensing. The response time is in the order of seconds, and the dynamic range is between 20%RH and 90%RH. The fabrication of the sensor head involved coating the HEC/PVDF thin film on a cladding-stripped fiber. The operating principle uses the changing refractive index of the film in response to changes in RH, which induces optical leakage as a function of RH. Ainhoa Gaston et al. [33] reported another humidity sensor based on a side-polished optical fiber with a PVA film. The response time is ~6 s. The sensitivity is 0.53 dB/%RH between 72%RH and 87%RH (1310 nm wavelength) or 0.66 dB/%RH between 55%RH and 85%RH (1550 nm wavelength). The dynamic range is 30%RH–90%RH. Likewise, dip coating the prepared fiber into a PVA solution creates the necessary thin-film coating. The sensing mechanism is seemingly evanescent wave-based, with water molecules inside the coating changing the output power due to water-induced absorption.

**Figure 8:**
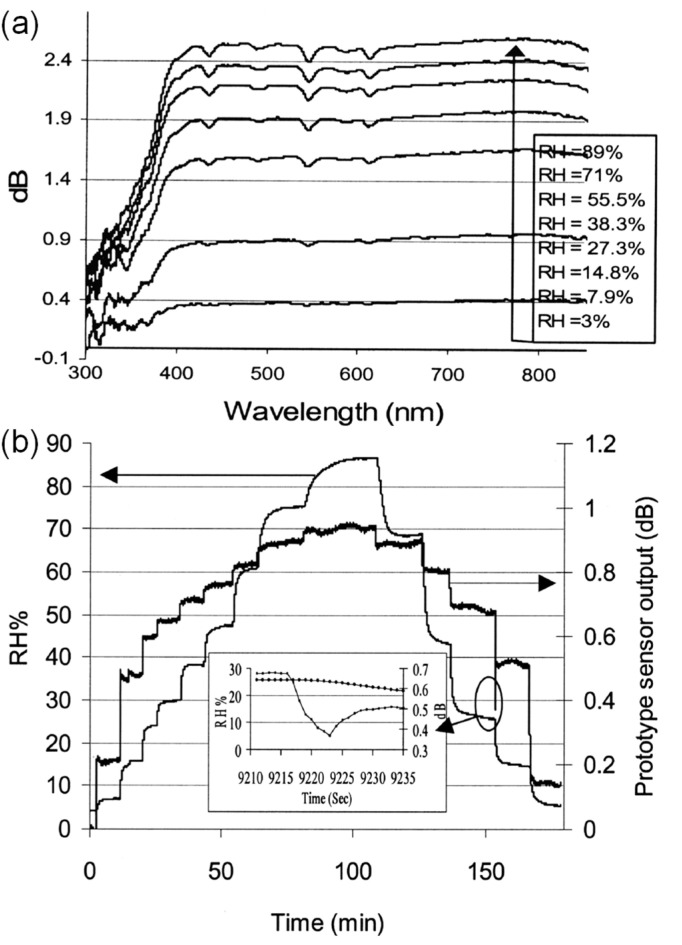
Point sensor. (**a**) Wavelength response to different RH. (**b**) Temporal responses of the humidity sensor. Adapted with permission from ref. [35]. Copyright 2004 *OSA*.

A highly sensitive humidity sensor using a TiO_2_ film overlay on side-polished optical fibers was presented by Alberto Alvarez-Herrero et al. [34]. The LOD is 0.2%RH. The response time is 10 s. The sensitivity is 460 pm/%RH between 1%RH and 15%RH, and the dynamic range is 1%RH–80%RH. The TiO_2_ film was deposited via electron-beam evaporation on a side-polished fiber held in a groove. Water molecules in the film change the coupling conditions between light propagating in the fiber and the substrate. The sensor works by monitoring the shift in resonant wavelength as a function of RH. Lina Xu et al. [35] (Figure 8) designed and tested a humidity sensor based on evanescent-wave scattering on the surface of a bent optical fiber. The response time is in the order of seconds. The sensitivity is 0.02 dB/%RH between 39%RH and 89%RH, and the dynamic range is 3%RH–89%RH. A side-polished optical fiber was bent and annealed. Then a silica sol solution was prepared and coated as a porous silica film on the surface of the fiber. With the ingress of water molecules, the light propagating through the fiber experiences scattering, the effect of which is observed at the output to give a relationship between output power and RH.

S. K. Shukla et al. [36] explored magnesium oxide films for humidity sensing with a U-shaped POF, with the aim of increasing the surface area for enhanced sensitivity. The sensitivity is 0.0005%/%RH between 65%RH and 80%RH, while the dynamic range is 5%RH–80%RH. The use of the sol-gel technique was adopted to coat MgO on a U-shaped optical fiber. The U-shaped optical fiber was dipped into the solution, dried, and gently heated. Water adsorption changes the refractive index of the cladding, and thus varying the local RH changes and optical confinement and thus output power. Khay Ming Tan et al. [37] reported the use of long-period fiber gratings (LPFGs) in conjunction with gelatin coating for high-humidity measurements. The LOD is 0.25%RH, and the sensitivity is 1.2 dB/%RH between 90%RH and 99%RH. The dynamic range is 90%RH–99%RH. A gelatin solution was prepared by mixing dry gelatin powder with distilled water. The LPFG was coated with gelatin after annealing for improved stability. The sensing mechanism is based on the absorption of water molecules to change the effective refractive index experienced by the propagating light and thus creates a change in the interference conditions and resonant wavelength.

**Figure 9:**
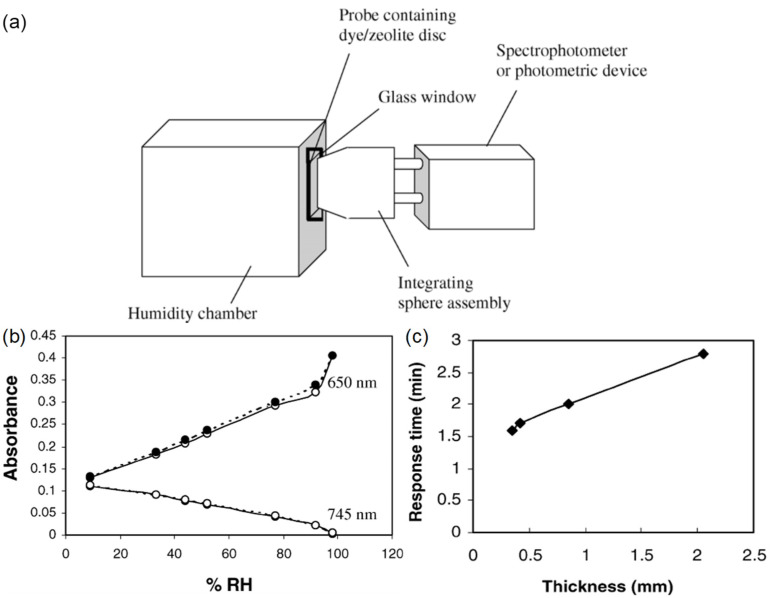
Point sensor. (**a**) Experimental setup. (**b**) Relationship between absorbance and RH. (**c**) Thickness-dependent response time between 9%RH and 98%RH at 650 nm wavelength. Adapted with permission from ref. [38]. Copyright 2005 *Elsevier*.

M. A. Zanjanchi et al. [38] (Figure 9) evaluated the use of methylene blue incorporated in zeolite for humidity measurements. The response time is 2 min. The sensitivity is 0.0025 dB/%RH between 9%RH and 92%RH, and the dynamic range is 9%RH–92%RH. Protonated mordenite was lightly ground and immersed in an aqueous solution of methylene blue, and the suspension was shaken in the dark. Finally, the sample was processed in an ultrasonic water bath, washed with hot deionized water, heat-dried, and finally compressed to form a disk. The working principle is based on the diffuse reflectance intensity change of 650 or 745 nm bands after exposure to RH. The overall color change depends on the protonation/deprotonation of dye molecules intercalated into the zeolite, which is governed by the dehydration/hydration of the zeolite. Teck L. Yeo et al. demonstrated a series of polyimide-coated fiber Bragg grating (FBG)-based humidity sensors. The first report [39] featured a response time of 18 min and a sensitivity of 5.6 pm/%RH between 23%RH and 97%RH. The dynamic range is 23%RH–97%RH. The second report [40] achieved a LOD of 2%RH. The response time is 25 min, and the sensitivity is 4.5 pm/%RH between 22%RH and 97%RH. The dynamic range is 22%RH–97%RH. In both cases, the gratings were dip-coated with a polyimide solution. The polymer coating functionalizes the FBG by transferring strain when it swells with increasing RH. The change in FBG length influences the observed Bragg wavelength.

**Figure 10:**
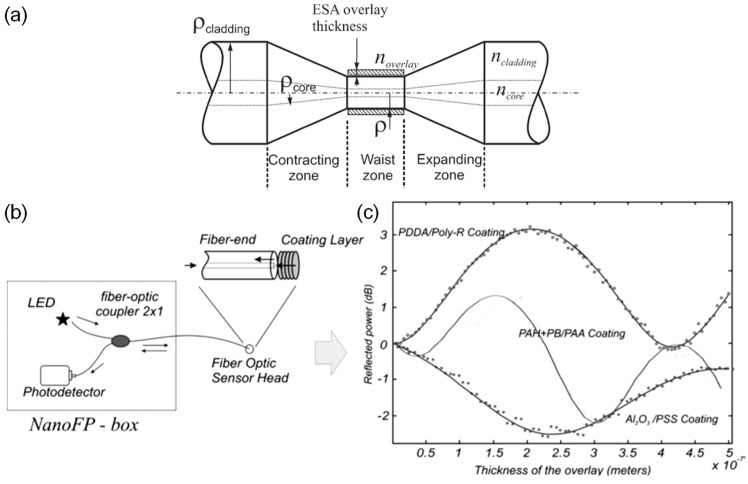
Point sensor. (**a**) Geometrical parameters of the fiber taper. (**b**) Experimental setup. (**c**) Reflected power as a function of overlay thickness. Adapted with permission from ref. [42]. Copyright 2006 *IEEE*.

Jesus M. Corres et al. [41] reported tapered fibers overlaid with a pairing of PDDA and poly-R (Dye R-478) nanofilms. The sensitivity is 0.8 dB/%RH between 75%RH and 90%RH, and the dynamic range is 75%RH–100%RH. The sensitivity without coating is 0.008 dB/%RH, which is two orders of magnitude lower. PDDA (polycation) and poly-R (polyanion) were prepared in a solution prior to dip coating. The absorption of water molecules in the coating changes its refractive index, which affects the guided modes and thus the output power. Jesus M. Corres et al. [42] (Figure 10) also presented design optimizations for taper-based humidity sensors, mainly focusing on coating thickness, taper dimensions, light source, and functional materials with different refractive indices.

Maximino Bedoya et al. [43] explored the concept of phase-sensitive fluorescence detection with a water-sensitive luminescent Ru(II) complex. The response time is 1.4 min, while the dynamic range spans 4%RH–100%RH. A PTFE disk was used as the substrate for the indicator solution to dry and form a luminescent membrane. The approach relies on modulating the intensity of the light source and using a lock-in amplifier to detect phase shifts (i.e., optical power, not electromagnetic waves) resulting from the emission lifetime. Different RHs affect the lifetime of the emission. Robert A. Barry et al. [44] investigated inverse opal hydrogels for humidity sensing. A response time of approximately 20 s was shown, alongside a tested range of 50.2%RH–80%RH. The solution containing the monomer was cross-linked by exposure to ultraviolet light (UV). An optical fiber in conjunction with a microscope was used to probe the hydrogel layer. The hydrogel swells, and the Fabry-Perot etalon changes in terms of optical path length, resulting in a shift in resonant wavelength in addition to a change in the reflectivity.

T. L. Yeo et al. [45] tested polyimide-coated FBG-based humidity sensors in concrete for practical applications. The response time is slow at ~2 h. The sensitivity is 3.7 pm/%RH between 23%RH and 96%RH, and the dynamic range is 23%RH–96%RH. As reported before, the operation relies on the polymer coating to swell with increasing RH, which stretches the FBG such that it alters the measured Bragg wavelength. In another test, T. L. Yeo et al. [46] demonstrated a shorter response time of 30 min. The sensitivity is 3 pm/%RH between 33%RH and 96%RH, and the dynamic range is 33%RH–96%RH.

**Figure 11:**
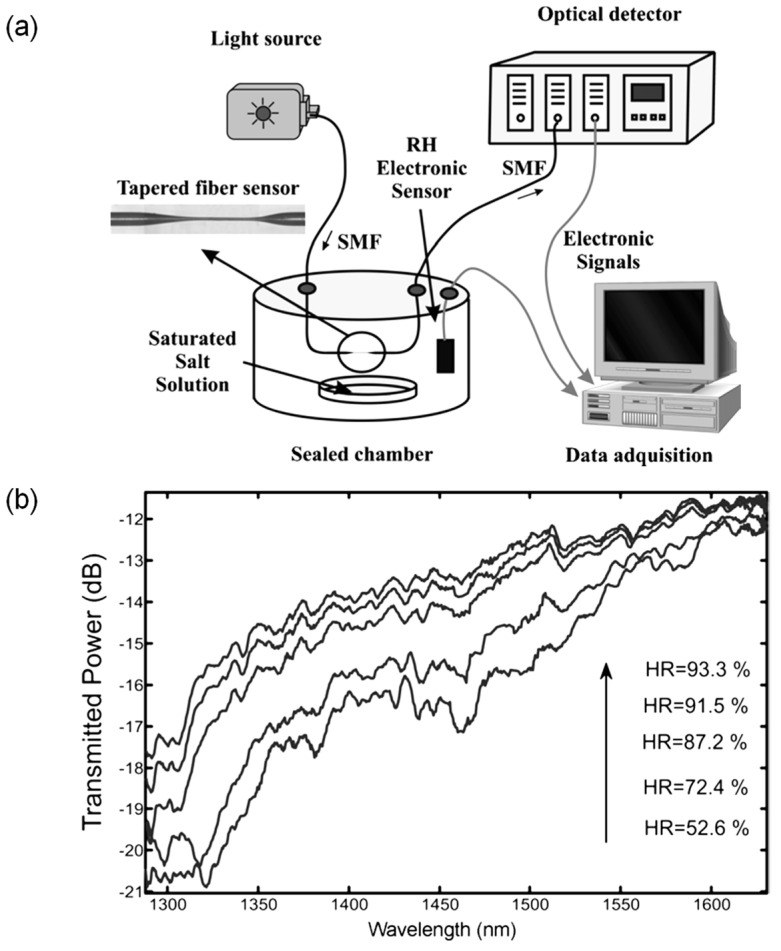
Point sensor. (**a**) Experimental setup. (**b**) Spectral response to different levels of RH. Adapted with permission from ref. [47]. Copyright 2007 *Elsevier*.

Optimization of an earlier work by Jesus M. Corres et al. [47] (Figure 11) was reported, which consists of a fiber tapered with PDDA/poly R-478 thickness tuning. The LOD can be as low as 1.3%RH, though the sensitivity varies monotonically. The response time is 300 ms, while the dynamic range is 75%RH–100%RH. Ignacio R. Matias et al. [48] expanded on the earlier work on fiber tapers by exploring hollow-core fibers (HCFs) with nanocoatings. It was found that HCFs offered a more consistent response at the expense of lower sensitivities. The response time for both designs is around 300 ms. The sensitivity of the fiber taper and HCF designs between 75%RH and 100%RH are 0.885 dB/%RH and 0.034 dB/%RH, respectively. The dynamic range is 75%RH–100%RH. The PDDA/poly R-478 coating was applied by dip coating. The HCF was spliced between two sections of multimode fiber (MMF) for light delivery and collection.

**Figure 12:**
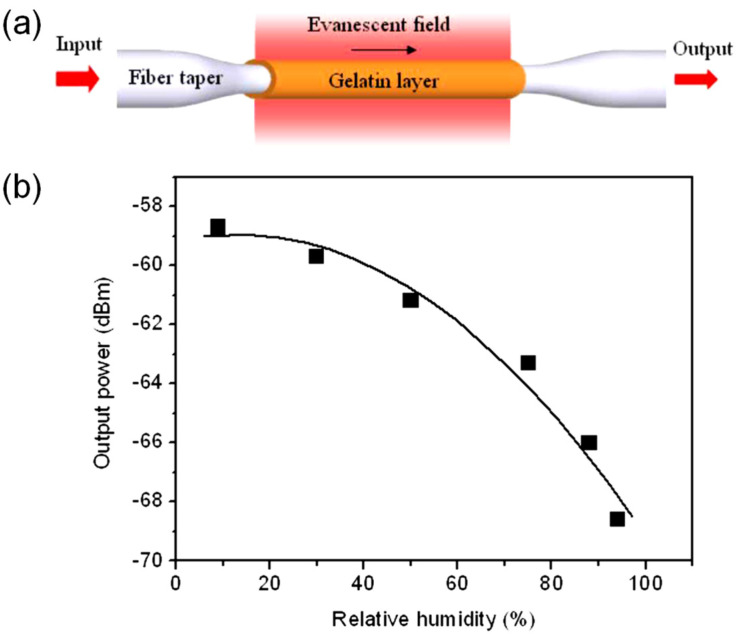
Point sensor. (**a**) Conceptual design of the sensor head. (**b**) Relationship between output power and RH. Adapted with permission from ref. [50]. Copyright 2008 *OSA*.

Fuxing Gu et al. [49] investigated bromothymol blue (BTB) doped poly(methyl methacrylate) (PMMA) nanowires for ultra-fast humidity responses. The response time is only 30 ms within a dynamic range of 35%RH–88%RH. The nanowires were fabricated by the direct drawing of solvated polymers. Orange light was coupled to the nanowire, and green light was collected from the nanowire by fiber tapers. The sensing mechanism is fluorescence, and the output power of fluorescence emission provides a readout of the RH. Lei Zhang et al. [50] (Figure 12) also demonstrated subwavelength-diameter fiber tapers coated with a gelatin film to sensitize the coated section. The response time is only 70 ms, and the dynamic range is 9%RH–94%RH. Powdered gelatin was dissolved in distilled water for dip coating the fiber taper. The operating method involves a change in water content in the coating that changes the effective refractive index, thus affecting the optical guidance. The output power is proportional to the RH.

A co-polyaniline cladding was explored by Anu Vijayan et al. [51], which involved coating a section of uncladded optical fiber. The response time is 8 s, and the dynamic range is 23%RH–88%RH. The sensing fiber was prepared by depositing Co nanoparticles dispersed in polyaniline over a cladding-stripped section of an MMF. The sensing mechanism relies on evanescent-field interaction with the coating to induce a refractive index change and thus optical leakage. Jesus M. Corres et al. [52] explored the use of SiO_2_-based fiber-tip polyelectrolyte coating. Since SiO_2_ can be tailored to exhibit either charge through different solutions, the functional coating only requires one material instead of two. The demonstrated response time is 150 ms. The sensitivity is 0.17 dB/%RH between 40%RH and 98%RH, and the dynamic range is 40%RH–98%. The fiber tip is dip-coated in two different solutions of SiO_2_ to build up the coating thickness layer-by-layer.

Jesus M. Corres et al. [53] used electrostatic self-assembled alumina (Al2O3) and poly(sodium 4-styrenesulfonate) as the functional cladding of an LPFG. A theoretical model of multilayer cylindrical waveguides based on coupled-mode theory was developed to explain and predict the measured results. A sensitivity of 440 pm/%RH was achieved between 50%RH and 75%RH, and the dynamic range is 50%RH–75%RH. The layer-by-layer assembly follows the usual protocol involving alternate dip coating, followed by a final wash and drying stage. The coating absorbs water molecules and changes the effective refractive index of the LPFG and thus induces a phase shift for interference and a shift in resonant wavelength. An intermediate layer of a higher-than-core refractive index was deployed to increase the evanescent field for higher sensitivity. T. Venugopalan et al. [54] developed a humidity sensor by combining LPFG with PVA. The response time is 80 s, within a dynamic range of 33%RH–97%RH. The PVA was made from mixing deionized water with PVA solution, then heated before the LPFG was pulled through the solution at a specific speed to control the coating thickness. The final stage was heating the coated fiber to dry fully. As with all evanescent wave-based LPFG designs, both the resonant wavelength and resonance extinction ratio changes with the increase in water ratio in the coating. This is due to changes in the effective refractive index, phase conditions, and water-induced propagation loss of probe light.

**Figure 13:**
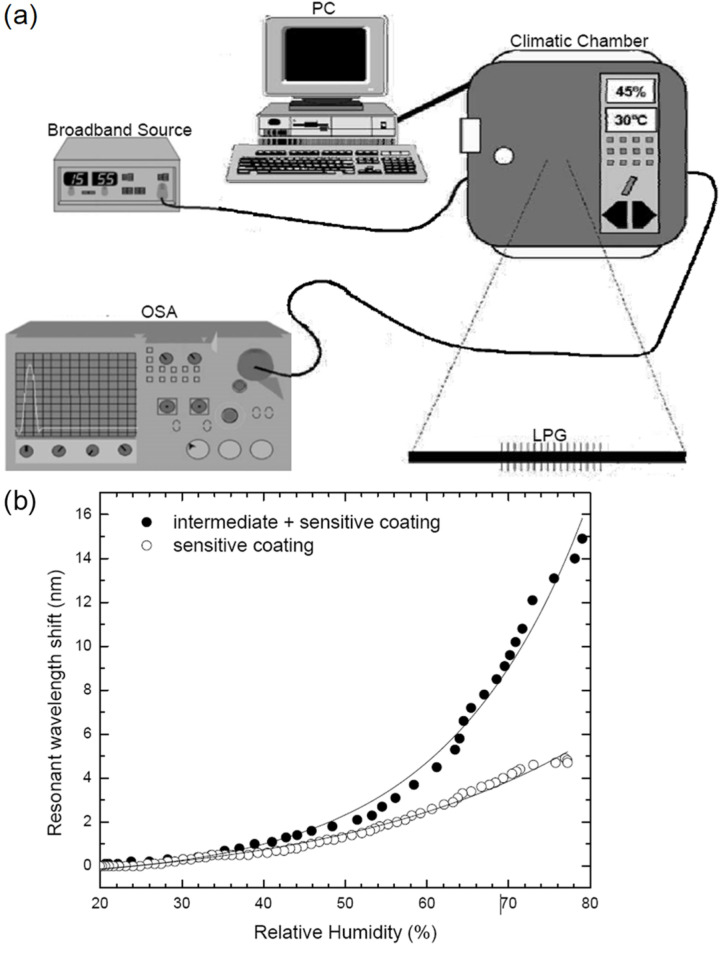
Point sensor. (**a**) Experimental setup. (**b**) Wavelength shift as a function of RH for two coating strategies. Adapted with permission from ref. [55]. Copyright 2009 *MDPI*.

The combination of LPFG and silica nanospheres was tested by Diana Viegas et al. [55] (Figure 13) to achieve a sensitivity improvement. The response time of the sensor is 100 ms, and its dynamic range is 20%RH–80%RH. A layer-by-layer assembly of polyelectrolyte was deposited by the ISAM method, consisting of PDDA, poly-R-478, and SM-30 SiO_2_-water colloid. SiO_2_ nanospheres are a porous hydrophilic material, and their water intake within the PDDA/poly-R-478 coating changes the effective refractive index seen by the guided modes, which changes the differential phase and shifts the resonant wavelength as a function of RH. S.K. Shukla et al. [56] explored the benefits of nano-crystalline zinc oxide film when coated onto a U-shaped optical fiber. The LOD is 0.45%RH, and the response time is 30 s. The sensitivity is 0.45 between 5%RH and 50%RH and 0.3 between 50%RH and 90%RH. The dynamic range is 5%RH–90%RH. The sensing fiber was dip-coated in the prepared precursor solution before being heated to realize the nano-crystalline ZnO cladding. The sensor operates by changing the optical confinement of the optical fiber with varying RH and thus the refractive index of the functional coating.

Xiujuan Yu et al. [57] investigated cascaded LPFG with hydrogel coatings and Fourier demodulation. The LOD is 0.39%RH, while the sensitivity is 9.9 × 10^−3^ a.u./%RH between 60%RH and 100%RH. The dynamic range is 25%RH–100%RH. Thin hydrogel films were overlaid on the LPFGs by dip coating. The sensing mechanism is based on differential phase, and thus a change in the resonant wavelength as well as its peak frequency after a Fourier transform. Yinping Miao et al. [58] reported a humidity sensor based on a tilted FBG with a humidity-responsive PVA coating to adjust the coupling conditions. The response time is 2 s. The sensitivity is 0.025 dB/%RH between 20%RH and 80%RH and 0.16 dB/%RH between 83%RH and 98%RH. The dynamic range is 20%RH–98%RH. Dip coating was employed to transfer the PVA onto the surface of the sensing fiber. Upon water absorption, the coating changes in refractive index, which affects the coupling to lossy cladding modes, and thus changes the measured output power.

**Figure 14:**
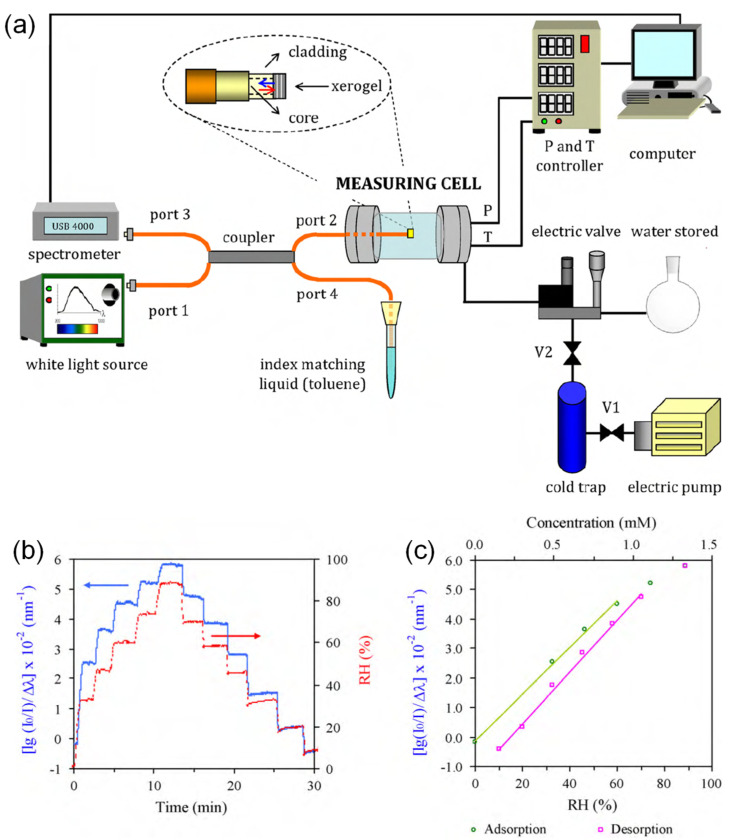
Point sensor. (**a**) Experimental setup. (**b**) Stepped RH cycle response. (**c**) Calibration curves in the range of 630–670 nm. Adapted with permission from ref. [59]. Copyright 2010 *Elsevier*.

A porous silica xerogel film on optical fiber was studied for its application for humidity measurement by Juncal Estella et al. [59] (Figure 14). The LOD is 4%RH, and the response time is 10 s. The sensitivity in the range of 630–670 nm wavelength is 0.079 a.u./nm/%RH between 0%RH and 60%RH (rising) and 0.088 a.u./nm/%RH between 10%RH and 70%RH (falling). The dynamic range is 4%RH–100%RH. The xerogel film preparation was performed by the sol-gel process and dip-coated onto an MMF. The sensing mechanism is based on the change in reflectivity measured using the MMF when water molecules are adsorbed on the silica xerogel film. Madhavi V. Fuke et al. [60] studied the effect of particle size of silver nanoparticles in polyaniline composite for humidity sensing. The optimum configuration leads to a response time of 30 s. The sensitivity can be as high as 28.78 mV/%RH between 5%RH and 95%RH, and the dynamic range is 5%RH–95%RH. A U-shaped POF was dip-coated with Ag-polyaniline nanocomposite to become sensitized to RH. The underlying principles involve a water-induced decrease in the refractive index of the coating, which improves its optical confinement. There is also a counteracting aspect involving the greater attenuation of light at higher levels of RH.

**Figure 15:**
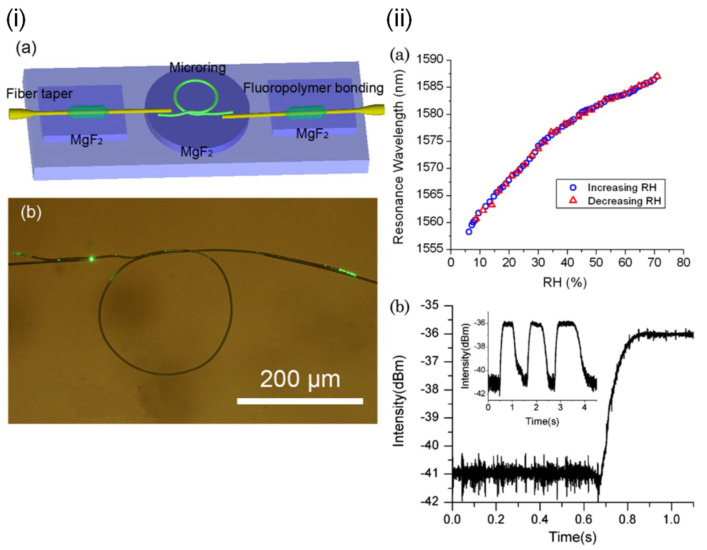
Point sensor. (**i**) (**a**) Conceptual design of sensor head. (**b**) Microscope image of the microfiber ring. (**ii**) (**a**) Resonant wavelength as a function of RH. (**b**) Temporal response. Adapted with permission from ref. [62]. Copyright 2011 *SPIE*.

Shohei Akita et al. [61] demonstrated a hetero-core optical fiber for humidity measurements. A response of time 400 ms was reported, within a dynamic range of 50%RH–93%RH. Poly-Glu and poly-Lys were used in the layer-by-layer assembly of the functional coating. With changing RH, the polyelectrolyte coating absorbs water molecules and changes its refractive index. This leads to a change in the total internal reflection conditions at the hetero-core junction, which affects the extent of light being recoupled into the core. Pan Wang et al. [62] (Figure 15) demonstrated microfiber rings constructed from polyacrylamide (PAM) microfibers as humidity-sensitive resonators. The response time is 120 ms, and the sensitivity is 490 pm/%RH between 5%RH and 71%RH. The dynamic range is 5%RH–71%RH. The PAM microfiber was fabricated by the direct drawing of a PAM solution and manipulated into a self-coupling loop (i.e., resonator) on a low-index MgF_2_ crystal substrate. Its stability on the substrate is provided by van der Waals force and electrostatic force. With rising RH, the PAM microfiber absorbs water molecules and swells, giving rise to an increased optical path length and thus a shift of the resonant wavelength.

Silica and polymer microfiber knot resonators were explored by Yu Wu et al. [63]. The LOD is 0.23%RH, and the response time is 500 ms. For the silica and polymer resonators, the sensitivity is 1.2 pm/%RH and 8.8 pm/%RH between 17%RH and 95%RH, respectively. The dynamic range is 17%RH–95%RH. The silica microfibers were fabricated by flame-heated taper-drawing of a single-mode fiber (SMF). The PMMA microfibers were manufactured through the direct drawing of solvated polymers. The operating principles are the same as the microfiber rings, where water ingress shifts the resonance spectrum. Andrii Buvailo et al. [64] presented research on thin polymer film-based surface acoustic wave (SAW) humidity sensors. The response time is 1.5 s, while the dynamic range is 2%RH–70%RH. The solution of PVA and poly(Nvinylpyrrolidone) (PVP) were deposited on a glass substrate via a photo-resist spinner. Transmission-mode Fourier transform infrared spectroscopy (FTIR) was used to measure infrared absorbance caused by OH-stretching vibrations of absorbed water molecules. The magnitude of absorption depends on the quantity of water in the film.

Biao Wang et al. [65] advanced the concept of CoCl_2_ and PVA-based humidity sensing with the addition of SiO_2_ into the composite solution. The response time is 2 min, while the dynamic range is 25%RH–65%RH. The sensor head was fabricated by dip coating a fiber tip into the composite solution followed by high-speed spinning to obtain a uniform coating. The working principle is simply water-induced absorption of the probe light, where the output power indicates the level of RH. Bobo Gu et al. [66] developed a humidity sensor based on an FBG inscribed in a thin-core fiber to form a modal interferometer. The LOD is 0.78%RH, and the response time is 2 s. The sensitivity is 84.3 pm/%RH (@20 °C) between 20%RH and 90%RH, and the dynamic range is 20%RH–90%RH. A modal Mach-Zehnder interferometer (MZI) was constructed by splicing a thin-core fiber to two standard optical fibers on each end. The thin-core fiber has an FBG written along its core for temperature reference and compensation. Layer-by-layer ISAM was carried on the sensing fiber with alternate immersion into P4VP•HCl (i.e., positively charged) and PVS (i.e., negatively charged). The sensor works by allowing water-induced swelling to change the external refractive index and thus amplify the path imbalance in the modal interferometer. This results in an optical power modulation when the two paths of light coherently recombine at the second SMF.

**Figure 16:**
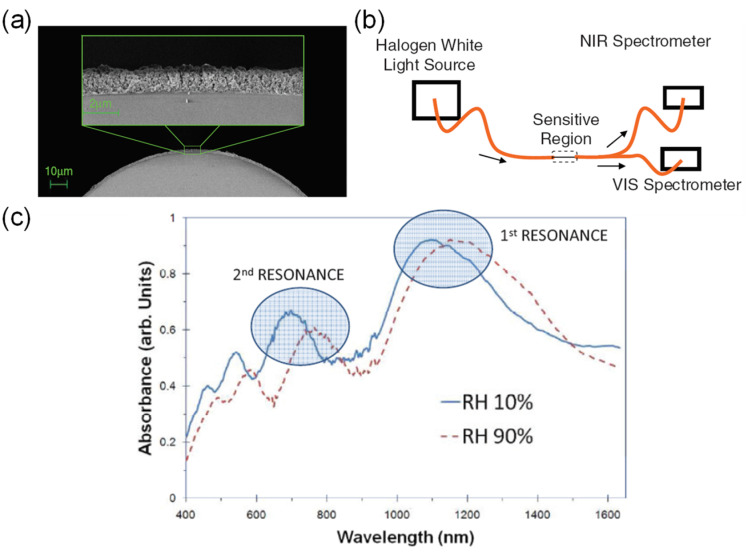
Point sensor. (**a**) SEM image of the coating on the fiber. (**b**) Experimental setup. (**c**) Spectral response to different RH levels. Adapted with permission from ref. [67]. Copyright 2011 *Elsevier*.

Lossy mode resonance (LMR) with titanium dioxide (TiO_2_) and PSS coating was demonstrated by C. R. Zamarreño et al. [67] (Figure 16). LMR is an attractive sensing mechanism because it is independent of the polarization of light and broadens the range of materials available for surface functionalization, including noble metals, metal oxides, and polymers. The response time is slow at 85 min. The sensitivity is 1.43 nm/%RH between 20%RH and 90%RH, and the dynamic range is 10%RH–90%RH. The ISAM method was employed for the coating of TiO_2_/PSS onto an MMF, facilitating a porous structure with a thickness comparable to that of the wavelength of the probe light. Upon absorbing water molecules, the change in the coating thickness and its refractive index leads to a change in the lossy resonance conditions, which shifts the resonance peaks to longer wavelengths. Matthew M. Hawkeye et al. [13] demonstrated a colorimetric humidity sensor based on TiO_2_ fabricated by using glancing angle deposition (GLAD). The LOD can be as low as 1%H with a dynamic range of 3–90%RH. The GLAD process enabled the fabrication of complex photonic crystal devices through a single step and was used in this work to coat a silicon or glass substrate with TiO_2_. As RH increases, water vapor penetrates the porous structure and condenses within the photonic crystal. As a result, the refractive index and optical path length increase. Both underlying effects are modified as a result, which is the Bragg resonance (i.e., band gap), and the FPI resonances (i.e., interference fringes) are shifted to longer wavelengths.

Ming-Yue Fu et al. [68] reported a humidity sensor based on an air-gap LPFG coated with calcium chloride. The sensitivity is 1.36 nm/%RH between 55%RH and 95%RH, and the dynamic range is 55%RH–95%RH. As no functional material is required, the sensor is relatively simple to construct. With rising RH, the adsorption of moisture increases the effective refractive index in the vicinity of the air gaps, which decreases the differential phase in the modal interferometer, and thus blue-shifts its resonant wavelength. Ruyang Xuan et al. [69] combined magnetic assembly and photo-polymerization to create a new photonic crystal film for humidity sensing. The response time is 10 min. The sensitivity is 1.8 nm/%RH between 11%RH and 100%RH, while the dynamic range is 11%RH–100%RH. Super-paramagnetic Fe_3_O_4_ colloids were prepared using a high-temperature hydrolysis reaction and then coated with a silica layer through a modified Stober process. Magnetic colloids and photo-initiator DMPA were dispersed in the mixture of hydrophilic poly(ethylene glycol) methacrylate (PEGMA), and cross-linker poly(ethylene glycol) diacrylate (PEGDA). The precuring mixture was sandwiched between a cover glass and a fluorinated glass slide, positioned above a NdFeB magnet and below a strong UV light source for photo-polymerization.

Wei Chang Wong et al. [70] reported a PVA-coated photonic crystal-based sensor for humidity measurements. The LOD varies between 0.04%RH and 0.5%RH between 50%RH and 90%RH. The response time is 300 ms. The sensitivity also varies from 0.04 nm/%RH to 0.15 nm/%RH between 50%RH and 90%RH. The dynamic range is 30%RH–90%RH. A range of PVA concentrations was carried out and prepared by dissolving PVA granules in deionized water before heating and drying. The coating was applied to the optical fiber via dip coating. The photonic crystal fiber (PCF) section forms a Michelson interferometer, while the thin-film coating acts as an FPI of higher fringe frequency. Upon absorption of water molecules, the functional coating changes its refractive index, which alters the optical path lengths and thus produces phase shifts detectable in the form of power modulations. Houhui Liang et al. [71] worked on a polarization-maintaining fiber loop mirror coated with PVA. The sensitivity is 220 pm/%RH between 20%RH and 85%RH. The PVA coating process follows the usual protocol. The change in the refractive index of the coating due to moisture ingress creates a phase shift between the two different polarization modes, which interfere and produce a power modulation as a function of RH.

**Figure 17:**
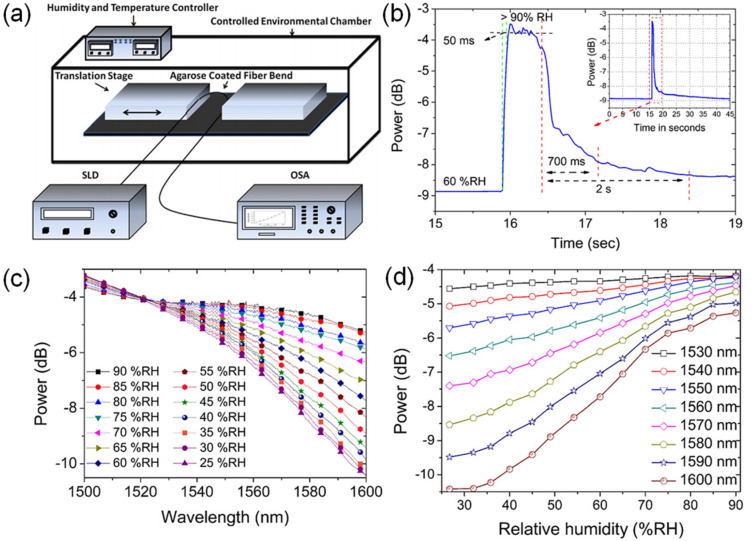
Point sensor. (**a**) Experimental setup. (**b**) Temporal response. (**c**) Relationship between wavelength and power for different RHs. (**d**) Relationship between power and RH for different wavelengths. Adapted with permission from ref. [72]. Copyright 2012 *Elsevier*.

A fiber-bend-based approach was taken by Jinesh Mathew et al. [72] (Figure 17), where an agarose coating applies humidity-dependent strain to the fiber. The response time is 50 ms. The sensitivity is 0.1 dB/%RH between 35%RH and 75%RH, while the dynamic range is 25%RH–90%RH. An agarose solution was deposited on an optical fiber before being arranged into a U-shape. With increasing RH, absorption of water increases the thickness of the coating, which changes the bend radius of the fiber. The consequent stress-induced change in the refractive index of the core and cladding leads to equal or near-equal refractive indices, which facilitate mode coupling and optical leakage. Li Han Chen et al. [73] developed an FPI with a thin layer of chitosan on the tip of an HCF section. The LOD is 1.68%RH, and the response time is 380 ms. The sensitivity is 130 pm/%RH between 20% and 95%RH, and the dynamic range is 20%RH–95%RH. A diaphragm on the tip of the HCF was made from an appropriate concentration of chitosan powder in an acetic acid solution. Water absorption by the coating changes its refractive index and optical path length, and consequently, the resonant wavelength as a function of RH.

Li Han Chen et al. [74] applied the same chitosan coating strategy to a polarization-maintaining fiber-based Sagnac interferometer. A detection of 2.04%RH was reported. The sensitivity is 81 pm/%RH between 20%RH and 95%RH, and the dynamic range is 20%RH–95%RH. The fiber was coated with chitosan, which reacts to water molecules and changes the differential phase between the two polarization modes to produce a power modulation. M. Y. Mohd Noor et al. [75] used an absorption spectroscopy technique with HCFs for measuring RH. The response time is 78 s, and the sensitivity is 3.02 mV/%RH between 0%RH and 90%RH. The dynamic range is 0%RH–90%RH. A section of HCF was spliced to the end of an SMF to enable air diffusion into its air holes for improved sensitivity.

A Boehmite nanorod and gold nanoparticle nanocomposite film was used by Priyank Mohan et al. [76] for local surface plasmon resonance (LSPR)-based humidity sensing. The response time was demonstrated to be 20 s. The mixed composite was spin-coated onto a glass substrate followed by heating to obtain the nanocomposite film. LSPR is sensitive to RH levels, as it affects the effective refractive index and thus resonance conditions. The coating was probed by a light source to monitor the shift in LSPR wavelength with changing RH, which featured a non-linear relationship. R. Aneesh et al. [77] presented a titanium dioxide nanoparticle-based optical fiber humidity sensor. The response time is 20 s. The sensitivity is 27.1 mV/%RH between 24%RH and 95%RH, and the dynamic range is 24%RH–95%RH. TiO_2_ nanoparticles were immobilized into a nanostructured sol-gel matrix and incorporated onto a cladding-removed optical fiber via dip coating. Absorption spectroscopy reveals the correlation between output power and RH.

**Figure 18:**
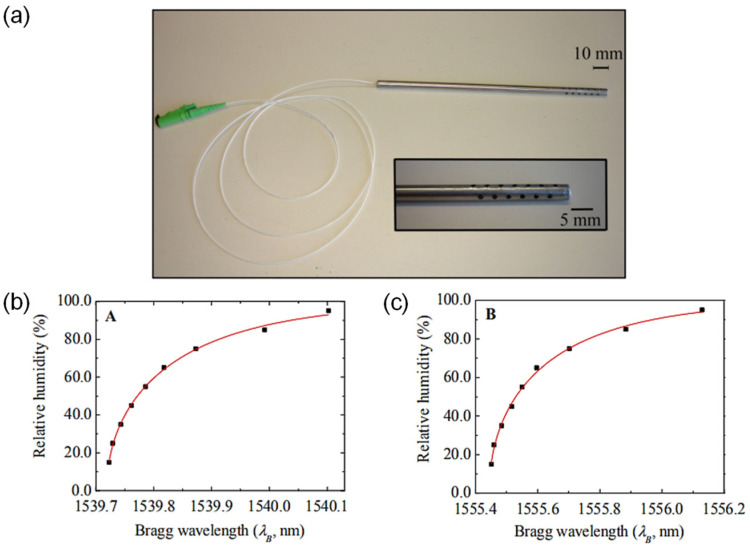
Point sensor. (**a**) Photograph of sensor head. Bragg wavelength dependence on RH for (**b**) FBG A, and (**c**) FBG B. Adapted with permission from ref. [78]. Copyright 2012 *MDPI*.

Sandra F. H. Correia et al. [78] (Figure 18) reported an FBG-based sensor with a coating of organo-silica hybrid material, Di-ureasil. The response time is 8.1 min, and the sensitivity is 22.2 pm/%RH between 70%RH and 95%RH. The dynamic range is 15–95%RH. The d-U(600) was deposited onto the FBG using a dip-coating system. The organo-silica-based material exhibits strong adhesion to the optical fiber and swells in the presence of water molecules. The expansion of the material also elongates the FBG, resulting in a change in its optical path length as well as its Bragg wavelength. Satyendra Singh et al. [79] conducted a comparative study on the surface morphologies of ferric oxide for humidity sensing. Owing to the non-linear relationship, on average, the sensitivity is around 1.5 µW/%RH between 10%RH and 70%RH and 0.35 µW/%RH between 70%RH and 90%RH. The dynamic range is 10%RH–90%RH. Aside from the surface morphological and structural investigations, the optical and thermal properties were studied using UV-visible absorption spectroscopy and differential scanning calorimetry (DSC) techniques. The sensor operates by using the changing refractive index of the thin film to provide a humidity-dependent reflectivity that can be measured using a simple light source. A Fabry-Perot etalon was avoided by using a wedge approach.

Tao Li et al. [80] developed a humidity sensor using a combination of tapered MMF, PVA coating, and FBG. The LOD is 1%RH, while the response time is 2 s. The sensitivity is 1.994 µW/%RH between 30%RH and 95%RH. The dynamic range is 30%RH–95%RH. The PVA coating was applied by using dip coating. The sensor operation can be described by evanescent wave-based absorption in the presence of water molecules. Increasing the RH decreases the refractive index of the coating, which suppresses light from escaping the core to be absorbed within the functional coating. The sensitivity is enhanced since the light passes through the taper twice from the back-reflection provided by the FBG. The temporal response of FBGs was investigated by W. Zhang et al. [81], with the aim of using chemical etching to reduce the fiber diameter and thus diffusion time of water molecules into a polymer optical fiber. The LOD is 0.12%RH, and the response time is 7 min for RH increase and 12 min for RH decrease. The sensitivity is 33.6 pm/%RH between 30%RH and 90%RH. The dynamic range is 30%RH–90%RH. The polymer FBG allows moisture to ingress and change its effective refractive index, thus modifying its optical path length and Bragg wavelength.

**Figure 19:**
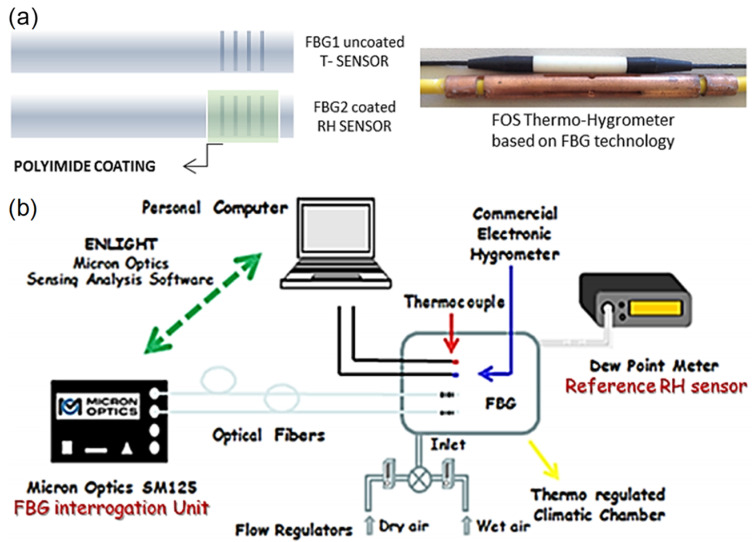
Point sensor. (**a**) Sensor head design and photograph. (**b**) Experimental setup. Adapted with permission from ref. [83]. Copyright 2014 *IOP Science*.

Yanjuan Liu et al. [82] introduced ZnO nanorods on tapered fiber to increase its sensitivity to humidity. The sensitivity is 0.014 /%RH between 60% and 95%, and the dynamic range is 10%RH–95%RH. ZnO nanorods were first synthesized in a colloidal solution before dip coating onto the fiber. The ZnO nanorod-covered fiber provides a sensitivity enhancement of almost 20 times, which is attributed to ZnO nanorods increasing the surface area for interactions and exhibiting dry air on its surface. The rapid surface adsorption of water molecules leads to a larger optical response. The effects of radiation on polyimide-coated FBGs were studied by A. Makovec et al. [83] (Figure 19). It was found that the sensing performance is immune to incrementally absorbed doses. However, the wavelength peaks undergo a radiation-induced shift. The sensitivity is 1.42 pm/%RH between 0%RH and 60%RH, and the dynamic range is 0%RH–60%RH. The FBG coated with hydrophilic polyimide swells with increasing RH due to water absorption. The strain transfers to the fiber, which changes the optical path length of the FBG, resulting in a shift in the Bragg wavelength.

Ginu Rajan et al. [84] etched a polymer FBG to reduce the response time and used a linear edge filter to convert wavelength shifts into intensity changes. The LOD is 0.5%RH, and the response time is 4.5 s. The sensitivity is 0.23 mV/%RH between 10%RH and 90%RH. The dynamic range is 10% and 90%RH. The response to humidity is facilitated by water-induced refractive index change and swelling, which change the optical path length of the FBG and thus instigate a shift in the Bragg wavelength. Jiali An et al. [85] investigated an SMF-MMF-SMF interferometric structure with PVA coating. The LOD is 1%RH. The sensitivity is 90 pm/%RH between 30%RH and 80%RH, or 0.5 dB/%RH between 30%RH and 80%RH. The dynamic range is 30%RH–80%RH. The section of multimode was spliced between the lengths of SMFs. The core of the MMF was coated with a layer of PVA. With increasing RH, water absorption of the coating changes the effective index of the cladding modes and generates a phase shift detectable as a power modulation.

Jinesh Mathew et al. [86] studied the impact of coating thickness on the sensitivity of a humidity sensor based on an agarose-coated PCF interferometer. The LOD is 0.07%RH, and the response time is 75 ms. The sensitivity is 64 pm/%RH between 25%RH and 40%RH and 137 pm/%RH between 40%RH and 90%RH. The dynamic range is 25%RH–90%RH. Coating with agarose was implemented out by pulling the PCF through a heated agarose solution. Similar to the interferometric structure demonstrated by Jiali An et al. [85], the SMF-PCF-SMF structural also acts as a modal MZI. Changes in the refractive index of the coating create a measurable differential phase.

A hybrid union of FBG and reflection-mode PCF interferometer was proposed and implemented by Jinesh Mathew et al. [87] for simultaneous measurement of RH and temperature. The response time is 1 s, and the dynamic range is 7%RH–47%RH. The FBG provides a temperature reading, while the PCF with its holes dip-filled with agarose gives the RH measurement capability through modal interferometry. Jing Hu et al. [88] worked on a planar humidity sensor based on cadmium telluride nanocrystals on porous silicon. The dynamic range is 12%RH–93%RH. The photoluminescence spectrum of CdTe and porous Si/CdTe were monitored using a spectrometer. It is hypothesized that increasing the RH eliminates surface traps and increases the lifetime of CdTe nanocavities, which heightens the emission.

Lourdes Alwis et al. [89] evaluated different designs of PVA- or polyimide-coated grating-based sensors and their performance for humidity sensing. PVA coatings offer a higher but non-linear sensitivity. Polyimide coatings offer a linear but lower sensitivity. The best possible LOD is 1.7%RH. The sensitivity is 600 pm/%RH between 40%RH and 85%RH, and the dynamic range is 20%RH–85%RH. Both PI and PVA swell with increasing RH, which changes their refractive indices. Since FBGs rely on the swell-induced strain, the variation in refractive index is more suitable for LPFGs, which is sensitive to the external refractive index. On the other hand, the swell effect can be used by both designs. Lourdes Alwis et al. [90] also demonstrated an LPFG-based PVA-coated humidity sensor using a Michelson interferometer with an inline mirror as the end-reflector. The LOD is 1.7%RH. The sensitivity is 600 pm/%RH between 40%RH AND 85%RH, and the dynamic range is 20%RH–85%RH. The LPFG acts as a modal interferometer and becomes sensitive to RH when coated with PVA, which enables the growth of the differential phase. The inline mirror reflects the light back for coherent recombination, resulting in a power modulation from the interference.

A no-core fiber structure was adopted by Li Xia et al. [91], with a combination of hydroxyethyl cellulose (HEC) and PVDF as the functional materials. The LOD is 2%RH, and the response time is 10 s. The sensitivity is 0.196 dB/%RH between 40%RH and 75%RH, and the dynamic range is 40%RH–75%RH. The gel material in the solution forms a stable system of transparent plastic, which gradually polymerizes under hydrolysis and polycondensation reactions to ultimately form a 3D mesh gel. The composite hydrogel absorbs water molecules to change its refractive index, which is employed by the modal MZI comprising a no-core fiber sectioned between two SMFs. Lourdes Alwis et al. [92] developed an analysis of polyimide-coated LPFGs for humidity measurements. The LOD is 0.8%RH, and the sensitivity is 100 pm/%RH between 20%RH and 80%RH. The dynamic range is 20%RH–80%RH. The PVA layer was dip-coated onto the fiber section containing the LPFG. The principle behind the design is the swelling of the coated polymer with increasing RH, which changes its refractive index, optical path length difference, and creates a shift in the resonant wavelength.

**Figure 20:**
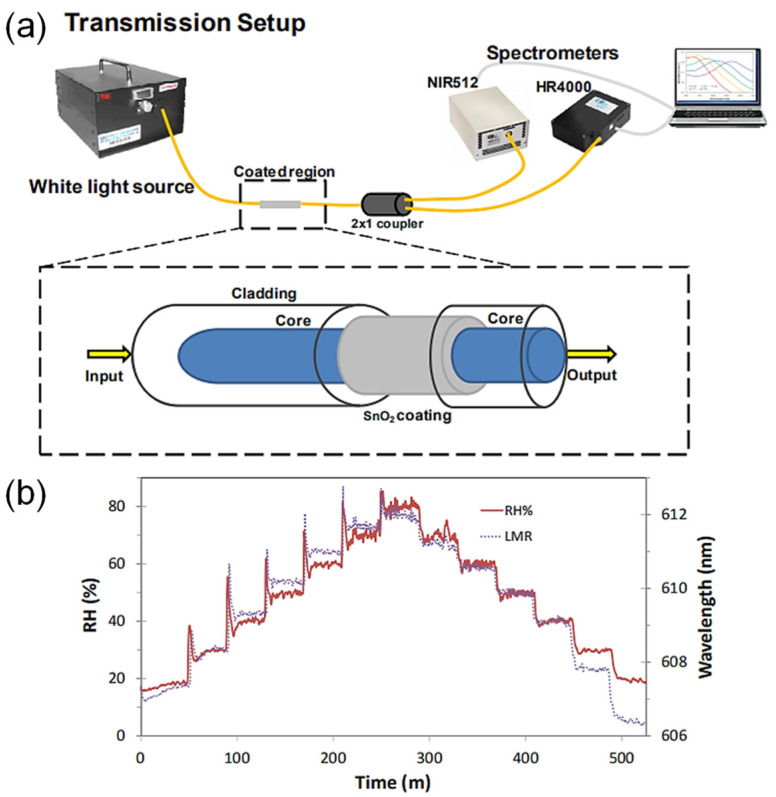
Point sensor. (**a**) Experimental setup and sensor head design. (**b**) Spectral response. Adapted with permission from ref. [93]. Copyright 2013 *SPIE*.

P. Sanchez et al. [93] (Figure 20) explored humidity sensing based on LMRs in optical fibers with tin oxide coating. The demonstrated sensitivity is 107 pm/%RH between 20%RH and 80%RH, within the dynamic range of 20%RH–80%RH. An ethanol-based solution of tin (IV) chloride pentahydrate was used as sol-gel precursor. A cladding-removed MMF was immersed into the precursor multiple times with an annealing process for each. Increasing RH increases the coating thickness and changes its refractive index, modifying the lossy resonance conditions. This consequently changes the resonant wavelength. S. S. Voznesenskiy et al. [94] developed integrated-optic humidity sensors using chitosan waveguide films. The response time is 2 s, and the dynamic range is 38%RH–40%RH. Thin chitosan films were deposited on substrates by spin coating. Unlike most sensors, the functional material is not used as a coating but as a waveguide. Light couples into the planar waveguide and out via prism couplers. Increasing the RH decreases the refractive index but increases the propagation loss. Therefore, the transmitted power provides a measure of the RH.

A combination of interior-coated PCF with LPFG was reported by Shijie Zheng et al. [95]. The sensitivity is 1.43 nm/%RH between 21%RH and 40%RH, and the dynamic range is 21%RH–40%RH. Two types of nanofilms were coated on the surface of air channels in the LPFG section using the ISAM method. The underlayer is polyallylamine hydrochloride (PAH+) and polyacrylic acid (PAA−), which is not responsive but increases the sensitivity by adjusting the refractive index of the surrounding material. The overlayer is alumina (Al_2_O_3_+) and poly-sodium 4-styrenesulfonate (PSS−), which is used for selective adsorption. Yinping Miao et al. [96] exploited two sensing mechanisms for a humidity sensor based on LPFG and agarose gel. The sensitivity is 114.7 pm/% RH between 25%RH–65%RH and 65%RH–96%RH. The dynamic range is 25%RH–96%RH. The LPFG is affected by both strain and refractive index changes as a result of RH variation in the agarose gel. This changes the optical path length difference and creates a shift in the resonant wavelength.

**Figure 21:**
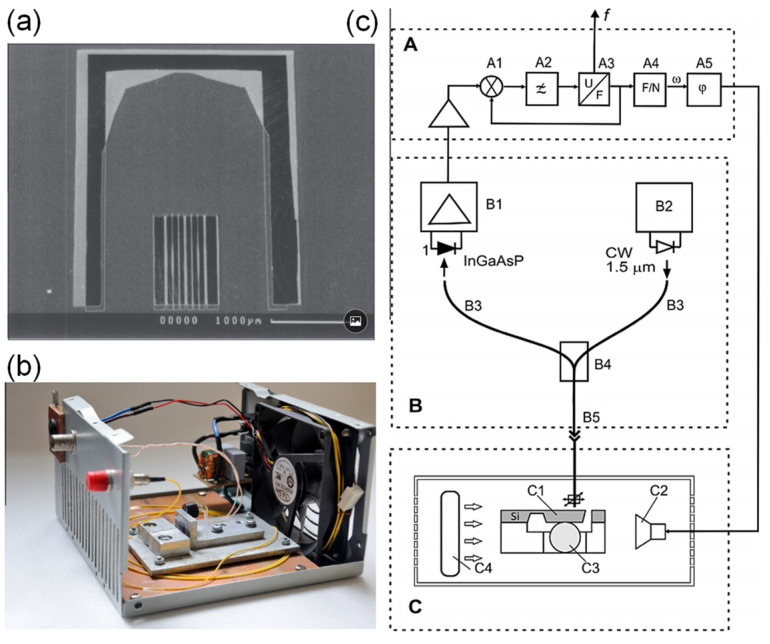
Point sensor. (**a**) SEM image of the sensor head. (**b**) Photograph of the sensing system. (**c**) Experimental setup: A—unit for phase lock: A1—synchronous detector with analog switches, A2—low pass filter, A3—voltage-controlled oscillator, A4—frequency divider, and A5—phase-shift module; B—optical part: B1—photodetector, B2—laser module, B3—optical fiber, B4–fiber coupler, and B5—optical connector; and C—sensitive element: C1—mechanical resonator, C2—miniature loudspeaker, C3—silica gel granule, and C4—mini-fan. Adapted with permission from ref. [98]. Copyright 2014 *Elsevier*.

Yun Cheng et al. [97] demonstrated a humidity probe based on an FPI at a fiber tip. The cavity comprises nanoporous titanium dioxide and silicon dioxide films. The sensitivity is 1.47 nm/%RH between 10%RH and 57%RH, and the dynamic range is 10.9%RH–92.8%RH. The dielectric thin films were fabricated by e-beam evaporation without ion-source assistance and exhibit columnar and porous structures. With the absorption of water molecules, the effective index of the cavity changes, resulting in a shift in the interference spectrum. Alexander V. Churenkov et al. [98] (Figure 21) used a silicon micromechanical resonant structure with an attached silica gel granule. The response time is 15 min. The sensitivity is 5 Hz/%RH between 4%RH and 75%RH, and the dynamic range is 4%RH–75%RH. The mechanical resonator was fabricated by anisotropic etching in a boron-doped silicon wafer. The top surface of the movable part reflected light to form an FPI. The bottom surface was bonded to a silica gel granule, which absorbs water molecules. With increasing RH, the mass of the moving part increases, which decreases the mechanical resonant frequency.

M. Batumalay et al. [99] applied agarose gel on etched POF. The LOD is 0.92%. The sensitivity is 0.0228 mV/%RH between 50%RH and 80%RH, and the dynamic range is 50%RH–80%RH. POF is porous, which absorbs moisture from the air to change its refractive index. A reduced-diameter POF can improve the migration rate of water molecules. Light propagating through the fiber interacts with the water molecules that lead to attenuation, which can be calibrated to the measured RH. Ni Hai-Bin et al. [100] reported multiple Fabry-Perot PCF-tip-based humidity sensors based on polystyrene opal films, silica inverse opal films, and composite photonic crystal films. Only the composite photonic crystal films exhibited linear sensitivities of 0.32 nm/%RH between 11%RH and 76%RH, or 13 intensity counts/%RH between 11%RH and 88%RH. The dynamic range is 12%RH–97%RH. The vertical self-assembly method was used to fabricate polystyrene opal films, which comprise a dried suspension of polystyrene microspheres. The sol-gel co-assembly method was applied to implement silica inverse opal films, which again uses a suspension of polystyrene microspheres. Composite photonic crystal films were made by infiltrating the gaps of the spheres in polystyrene opal films with silica precursor gel before heating and drying. The sensors operate by absorbing water molecules to swell up, which changes the optical path length and thus shifts the reflectance spectrum.

Iron titanium oxide was tested for humidity-sensitive properties by Nidhi Verma et al. [101]. The dynamic range is 5%RH–95%RH. A thin film of FeTiO_3_ gel was deposited on an isosceles prism and annealed to form the functional coating. The coating responds to moisture in the air by changing its refractive index with the intake of water molecules. The reflected optical power was collected to provide a readout of the RH. Branislav Korenko et al. [102] created a temperature-compensated humidity sensor based on FBG-inscribed birefringent fiber. The sensitivity is 2.6 pm/%RH (@20 °C) between 50%RH and 95%RH, and the dynamic range is 50%RH–95%RH. RH variations shift the polarization-dependent Bragg wavelengths simultaneously, and thus one wavelength peak can be tracked to provide a readout of RH. Temperature affects the shift of the two Bragg wavelengths differently, and thus the separation between the two wavelength peaks is used to indicate the temperature.

Chujia Huang et al. [103] developed a fiber-tip humidity probe by constructing an FPI based on porous Al_2_O_3_ film. The LOD is 0.65%RH, and the response time is 18 min. The sensitivity is 310 pm/%RH between 20%RH and 90%RH, and the dynamic range is 20%RH–90%RH. Porous anodic alumina (PAA) was fabricated starting from high purity aluminum, which was electrochemically polished and anodized before removal in phosphoric acid. The PAA was attached to the tip of an optical fiber using adhesive. The micro-pores readily absorb water molecules due to the capillary condensation effect, albeit they are easily saturated at higher RH. The change in the effective refractive index in the etalon produces a shift in the resonance spectrum as a function of RH. Z. Harith et al. [104] compared tapered silica SMF and POF coated with ZnO nanostructures. Since the tapered diameter of the silica taper was not revealed, and the two experiments used different light sources with possibly different coupling methods (e.g., core or cladding), it is not possible to confirm the conclusion that the tapered POF comprehensively offers higher sensitivity. The best sensitivity is 0.176 mV/%RH between 50%RH and 70%RH, while the dynamic range is 50%RH–70%RH. The SMF was flame-pulled while the POF was chemically etched. A sol-gel immersion method was employed to coat the fibers with ZnO nanostructures. With increasing RH, the functional coating adsorbs water molecules and increases its effective refractive index, which induces more optical leakage from the fiber.

**Figure 22:**
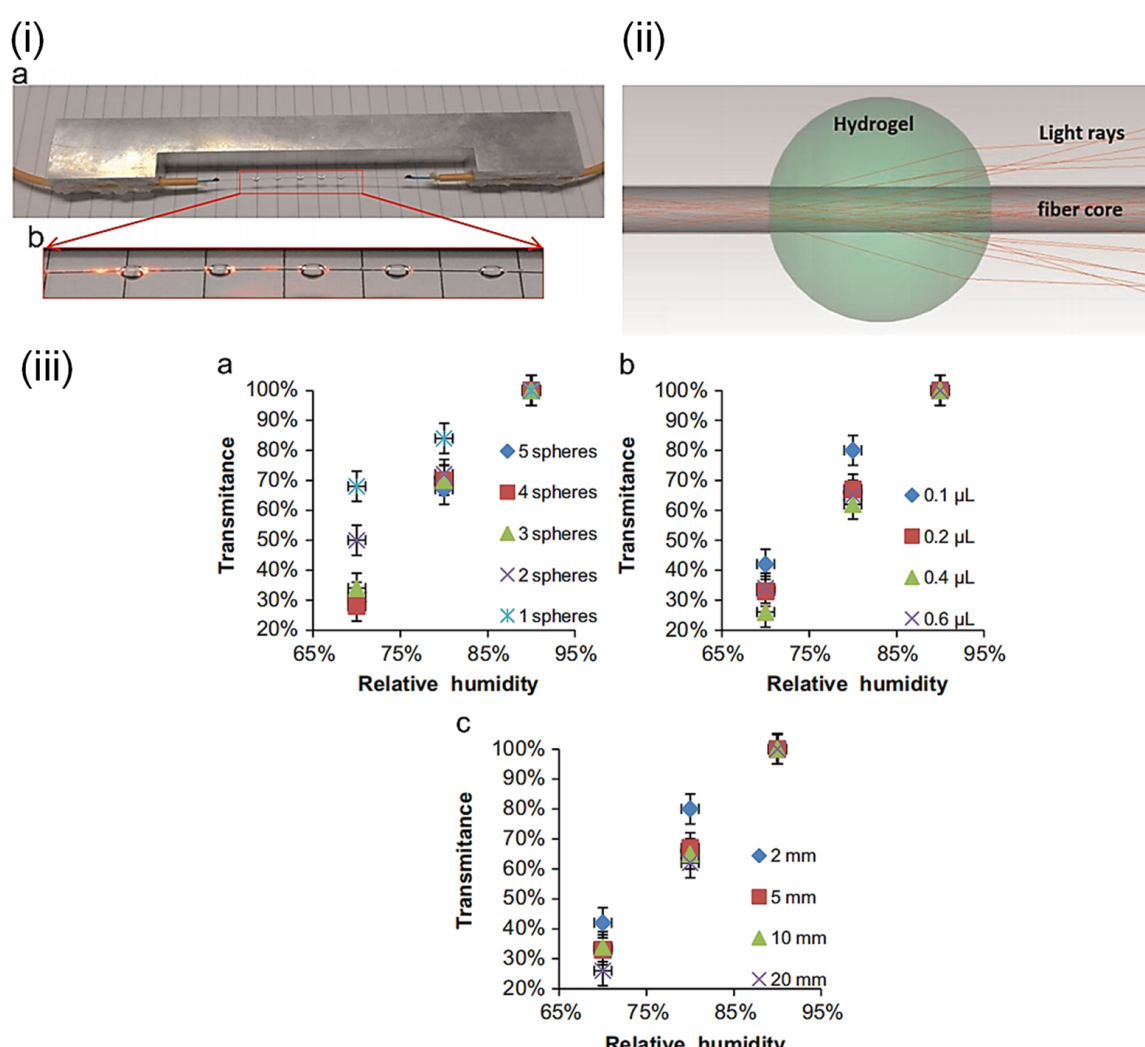
Point sensor. (**i**) Photograph of the sensor head array, (**a**) The sensor head; (**b**) magnified view of the five spheres with light input from the left side. Bright spots away from hydrogel spheres along fiber core are due to slight damage to the core surface during fabrication. (**ii**) Conceptual design of each sensing node. (**iii**) Influence of (**a**) sphere number, (**b**) size, and (**c**) separation space on the optical response. Adapted with permission from ref. [105]. Copyright 2015 *Elsevier*.

An intensity-based humidity sensor was conceived by Zhi Feng Zhang et al. [105] (Figure 22), which uses mini-hydrogel spheres on bare fiber cores. A ray-tracing simulation was carried out to study the influence of refractive index, size, quantity, and separation distance of the spheres. The sensitivity is 3.9%/%RH between 70%RH and 90%RH, and the dynamic range is 70%RH–90%RH. The hydrogen spheres were deposited on the bare fiber core by a micropipette before UV-induced crosslinking. Increasing the RH lowers the effective refractive index of the hydrogel, allowing better confinement of light and increased transmission. A. J. Swanson et al. [106] developed a synthesized polyetherimide coating for FBG-based humidity sensors, and it was compared with commercially available polymer coatings. The response time of the synthesized coating is 4.4 min. The sensitivity is 5.29 pm/%RH between 20%RH and 95%RH, and the dynamic range is 20%RH–95%RH. The sensing mechanism involves swelling-induced straining of the FBG, which changes its optical path length and Bragg wavelength.

Aitor Urrutia et al. [107] performed concurrent humidity and temperature sensing by using an LPFG partially coated with PAH/PAA. The sensitivity is 63.23 pm/%RH between 20%RH and 80%RH, and the dynamic range is 20%RH–80%RH. The PAH/PAA coating was fabricated with the layer-by-layer assembly technique (i.e., ISAM). The functional coating absorbs water molecules from the air and changes its refractive index, forcing a change in the optical path length of the cladding mode, and thus a spectrum shift. A modal MZI using a zinc oxide nanowires coating was realized by Asiah Lokman et al. [108]. The response time is 5 s. The sensitivity is 20.5 pm/%RH between 35%RH and 60%RH, and the dynamic range is 35%RH–60%RH. The dumbbell-shaped sensing fiber region was fabricated by a fusion splicer. The functional coating provides a surface for the adsorption of water molecules, and it changes the effective refractive index of the cladding mode for wavelength-shift measurements.

Getinet Woyessa et al. [109] reported a temperature-insensitive and hysteresis-free microstructured FBG made from PMMA for humidity sensing. The sensitivity is 35 pm/%RH between 10%RH and 90%RH, with a dynamic range of 10%RH–90%RH. The air holes appear to allow water vapor to reach the FBG in the core while maintaining a single-mode operation for a cleaner spectrum. The accumulation of water molecules changes the effective refractive index of the FBG region, alters the optical path length, and thus shifts the Bragg wavelength. HabibahMohamed et al. [110] implemented a humidity sensor based on tapered fiber coated with GO. The sensitivity is 0.0606 dB/%RH between 40%RH and 60%RH, and the dynamic range is 40%RH–60%RH. The fiber taper was manufactured using the common flame-brushing technique. The GO was prepared by using simplified Hummer’s method, then mixed with PVA and dropped onto the fiber. The GO coating adsorbs water molecules and changes its refractive index. This results in diffusive optical leakage from the fiber taper to the coating, where water-induced absorption modulates the transmitted power as a function of RH.

**Figure 23:**
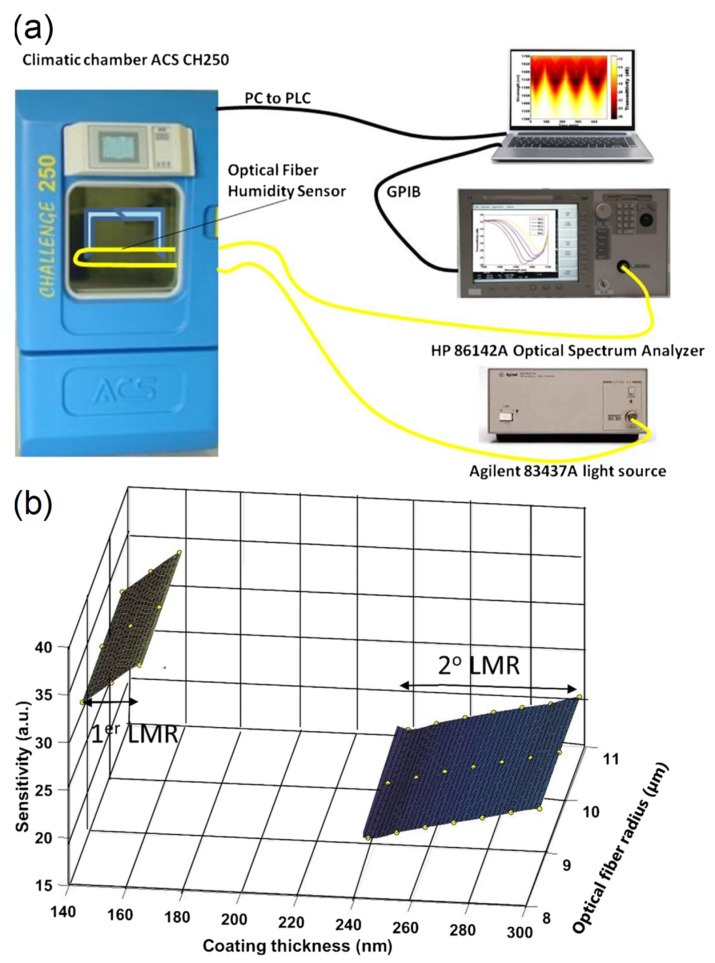
Point sensor. (**a**) Experimental setup. (**b**) Simulated sensitivity as a function of different diameters. Adapted with permission from ref. [111]. Copyright 2016 *Elsevier*.

J. Ascorbe et al. [111] (Figure 23) worked on a humidity sensor based on cladding-etched optical fiber and LMR with a layer of tin oxide. The demonstrated response time is 1.5 s. The sensitivity is 1.9 nm/%RH between 20%RH and 90%RH, within a dynamic range of 20%RH–90%RH. The tapered etching of the cladding was performed using hydrofluoric acid, and the coating was sputtered onto the fiber. The nano-coating comprising SnO_2_ produces the LMR, whose effective refractive index is sensitive to RH. A microfiber knot resonator with a PVA overlay was developed by Jong Cheol Shin et al. [112]. The sensitivity is 0.87 /um/%RH between 20%RH and 80%RH, with a dynamic range of 20%RH–80%RH. After the microfiber was arranged in a microknot on an MgF_2_ substrate, the PVA overlay was deposited using the spin-coating technique. The microfiber allows a significant portion of the evanescent field to interact with the PVA. The PVA coating changes its refractive index with varying RH and thus alters the effective refractive index seen by the guided mode(s). Such changes affect the resonant wavelength, which can be tracked as a function of RH.

Lu Shili et al. [113] reported a PVA-coated MZI based on two waist-enlarged fiber tapers. The response time is 3 s. The sensitivity is 98.8 pm/%RH between 40%RH and 90%RH, and the dynamic range is 40%RH–90%RH. The modal interferometer relies on the PVA coating to alter the effective refractive index seen by light, which creates a phase-shift-induced power modulation. Mahdiar Ghadiry et al. [114] used GO and nano-Anatase TiO_2_ to create a differential humidity sensor based on two different polymer waveguides. The response time is 700 ms. The sensitivity is 0.47 dB/%RH between 40%RH and 90%RH, and the dynamic range is 35%RH–98%RH. The GO was fabricated using a variant of Hummer’s method, which used deionized water as the solvent. TiO_2_ nanoparticles were synthesized in ethanol. Two SU8 polymer waveguides were fabricated on a SiO_2_ substrate, each with two layers being undercladding (i.e., via electrospinning followed by UV exposure and contact photolithography) and core layer (i.e., via spin coating). Fiber arrays were aligned to the channels for integration. The two different coatings were drop-casted onto the two waveguides and UV cured. In terms of operation, increasing RH leads to an increase in coating refractive index of the TiO_2_-coated waveguide, which reduces optical confinement and increases water-absorption-induced optical loss at the interface. The opposite effects occur for the graphene-oxide-coated waveguide due to increasing interlayer distance with increasing RH. The output signal is taken from the difference between the waveguide transmissions to obtain the maximum possible sensitivity.

**Figure 24:**
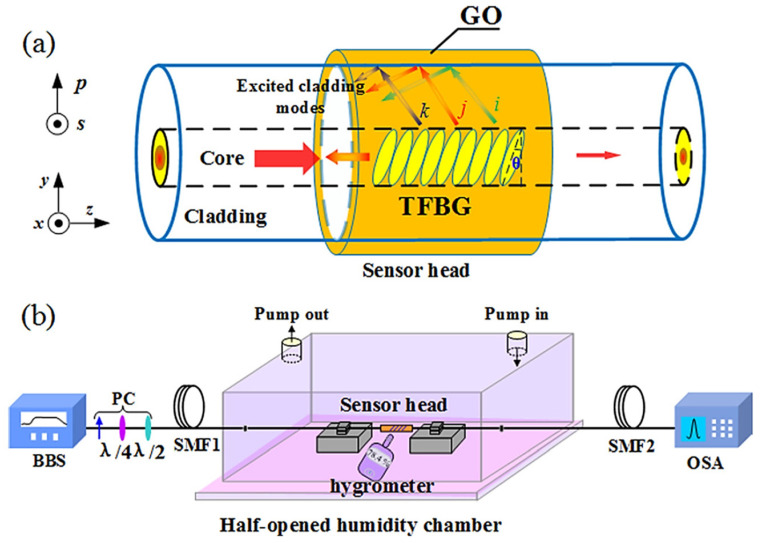
Point sensor. (**a**) Schematic of the sensing fiber. (**b**) Experimental setup. Adapted with permission from ref. [116]. Copyright 2016 *AIP*.

A GO PVA composite film was developed by Youqing Wang et al. [115], which is similar to the work performed by Lu Shili et al. [113], except with the added GO. The sensitivity is 0.193 dB/%RH between 30%RH and 75%RH, and the dynamic range is 25%RH–80%RH. Likewise, the dual waist enlargement was implemented by a fusion splicer. The composite GO/PVA coating acts as the actuator to influence the effective index of the sensing region, which is a path of the modal MZI. Light couples into the cladding mode upon entering the first waist-enlarged region and beats at the second one to produce interference-induced power modulation at the core. Youqing Wang et al. [116] (Figure 24) also used GO in conjunction with a tilted FBG for a different humidity sensor. The LOD is 0.7%RH, and the response time is 1 s. The sensitivity is 0.129 dB/%RH between 10%RH and 80%RH, and the dynamic range is 10%RH–80%RH. The tilted FBG section was dip-coated in GO solution. The tilted FBG excites cladding modes, which are reflected at the functional coating interface with the fiber, and the modes interfere back in the core. The RH-dependent refractive index of the coating modulates the phase difference between the modes and thus changes the resonant wavelength accordingly.

Yunhan Luo et al. [117] side-polished a fiber and applied a tungsten disulfide film for humidity sensing. The LOD is 0.475%RH, while the response time is 1 s. The sensitivity is 0.1213 dB/%RH between 35%RH and 85%RH, and the dynamic range is 35%RH–85%RH. A commercial WS_2_ alcohol suspension is treated by ultrasonication before being dropped into a basin containing the side-polished fiber. As the RH increases, more water molecules are adsorbed on the WS2 layer with moderate adsorption energies and a moderate degree of charge transfer. Larger amounts of electrons are transferred from WS2 to H_2_O, which reduces the conductivity and light absorption. As a result, the transmission of the fiber is proportional to the RH. George Y. Chen et al. [118] exploited a U-shaped optical microfiber with PDDA/PSS coating for ultra-fast humidity sensing. The LOD is 1.6%RH, while the response time is only 3 ms. The sensitivity is 2.7%/%RH between 60%RH and 80%RH, and the dynamic range is 0%RH–100%RH. The fiber taper is dip-coated with alternating layers of PDDA and PSS to build up the optically homogeneous humidity-sensitive coating. The U-shape allows optical leakage that enhances the evanescent field. With increasing RH, the polyelectrolyte coating swells and increases the water-induced absorption and scattering of light. Owing to the thin coating and thin fiber, the response time is minimized.

A lithium chloride film coated on the end-face of an optical fiber was used by Bao-Kai Zhang et al. [119] to quantify RH. The response time is 5 s, and the sensitivity is 10 nW/%RH between 11%RH and 75%RH. The dynamic range is 11%RH–75%RH. LiCl granules were mixed with deionized water to form a solution used for immersing the fiber tip. With the absorption of water molecules, the refractive index of the coating changes, which changes the reflectivity of light. A similar setup operates in parallel to provide a reference to the output power of the laser source. Carlo Massaroni et al. [120] used FBGs for simultaneous monitoring of temperature and RH. The response time is long, at 20 min. The sensitivity is 140 pm/%RH between 10%RH and 95%RH, and the dynamic range is 10%RH–95%RH. FBGs with different center wavelengths were coated with agar or agarose as the humidity-activated strain transducer. It was observed that the larger coverage of agar provided the largest wavelength shift, while agarose induced the least wavelength shift. However, the larger the coating area, the slower the response.

**Figure 25:**
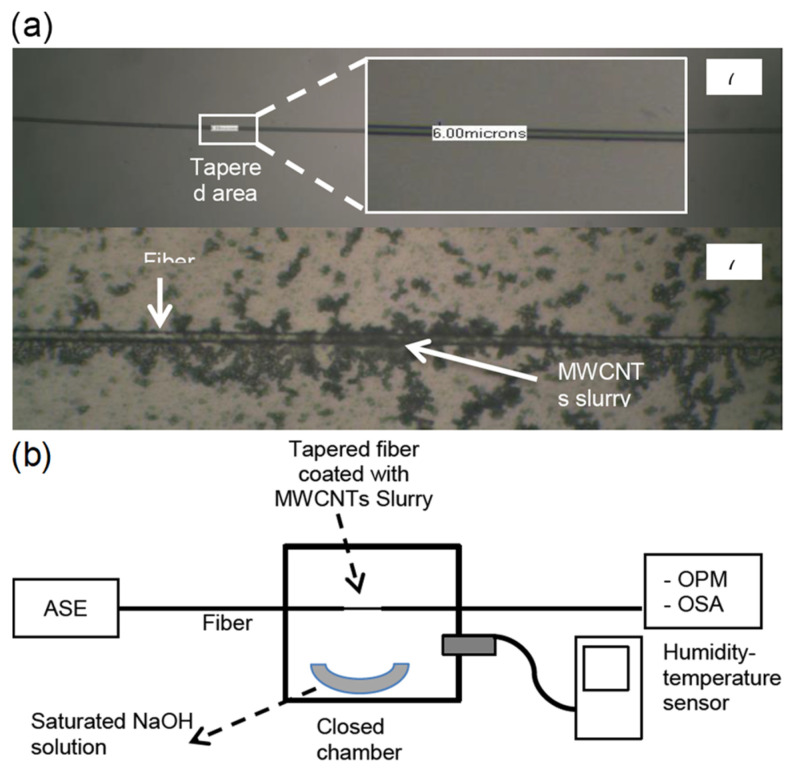
Point sensor. (**a**) Top: bare tapered fiber. (**b**) Droplet of MWCNTs-acetone on the tapered optical fiber. (**b**) Experimental setup. Adapted with permission from ref. [121]. Copyright 2017 *IASE*.

Tapered fiber coated with multiwalled carbon nanotubes slurry was explored by Habibah Mohamed et al. [121] (Figure 25). The LOD is 0.177%RH. The sensitivity is 5.17 μW/%RH between 45%RH and 80%RH, and the dynamic range is 45%RH–80%RH. A 3D printing filament used multiwalled carbon nanotubes dispersed in acrylonitrile butadiene styrene resin, and the result was extruded and ultrasonic processed to dissolve it in a suspension, which was used to drop onto the fiber taper for coating. The transmitted power was seen to increase with increasing RH, and the central wavelength decreased with increasing RH. Hamid E. Limodehi et al. [122] relied on the rate of vapor condensation to indicate the RH based on surface plasmon resonance (SPR) to detect changes in the refractive index. The LOD is 3%RH. The sensitivity is 0.11 mV/s/%RH between 15%RH and 55%RH, and the dynamic range is 10%RH–85%RH. A thin layer of gold was sputtered onto an MMF, and the monitoring of the plasmonic loss provides a measure of the RH. With this technique, a complex algorithm may be required to calculate fast-varying RH due to the observation of the rate of condensation.

Jia Shi et al. [123] incorporated a humidity-responsive Fabry-Perot etalon into a ring fiber laser. The LOD is 5%RH, and the response time is 72 ms. The sensitivity is 0.202 dB/%RH between 25%RH and 95%RH, and the dynamic range is 25%RH–95%RH. To form the FPI, a hollow Pyrex glass tube is sandwiched between an SMF and a silicon diaphragm coated with agarose. Upon absorbing water molecules, the reflectivity of the humidity-responsive agarose is modulated, which translates to a change in the output power. Kasun P. W. Dissanayake et al. [124] coated LPFG with GO for humidity sensing. The sensitivity is 0.15 dB/%RH between 60%RH and 95%RH, and the dynamic range is 60%RH–95%RH. An improved Hummer’s method was used to synthesize the GO, and an aqueous dispersion was used to coat the LPFG. The GO changes the local refractive index with varying RH, which affects the optical path length difference between the modes in the LPFG, and results in both resonant wavelength and intensity changes as a function of RH. Getinet Woyessa et al. [125] developed a Zeonex/PMMA microstructured polymer optical fiber (mPOF) Bragg grating sensor for concurrent RH and temperature monitoring. The exclusive reliance on polymer fiber gives rise to greater flexibility and paves the way for specific applications involving tight bends. The LOD varies from 0.4%RH to 1.1%RH between 10%RH and 90%RH. The average sensitivity is approximately 10.9 pm/%RH between 10%RH and 90%RH. The dynamic range is 10%RH–90%RH.

A fluorescent resonator humidity sensor was developed by Katalin Szendrei et al. [126] based on humidity-swellable antimony phosphate nanosheets embedding dye nanospheres. The dynamic range was demonstrated as 15%RH–90%RH, with no clear trends. Alternate layers of SiO_2_ and TiO_2_ nanoparticles were deposited from stable colloidal precursor suspensions to create the first multilayer. Subsequently, a suspension of H_3_Sb_3_P_2_O_14_ exfoliated nanosheets humidity-responsive optical cavity was spin-casted onto it to form the optical cavity. Then, a monolayer of light-emitting polystyrene nanospheres was deposited and covered by a new layer of H_3_Sb_3_P_2_O_14_. Swelling of the nanosheet layer by increasing the RH shifts the spatial and spectral positions of the optical cavity resonances relative to those of the emission band of the dyes. The result is either a fluorescent turn-off or a turn-on effect with changing RH. Weijia Wang et al. [127] introduced pore-foaming agent doped polyimides to FBGs for humidity sensing. The additive of lithium chloride to polyimide improved the sensitivity almost three-fold. The response time is 33 s, and the sensitivity is 1.71 pm/%RH between 20%RH and 88%RH. The dynamic range is 20%RH–88%RH. FBGs were dip-coated with pore-foaming agent doped polyimide before an annealing process to obtain a porous structure. With increasing RH, water diffusion through the FBG changes its effective refractive index, which affects its optical path length and thus Bragg wavelength.

**Figure 26:**
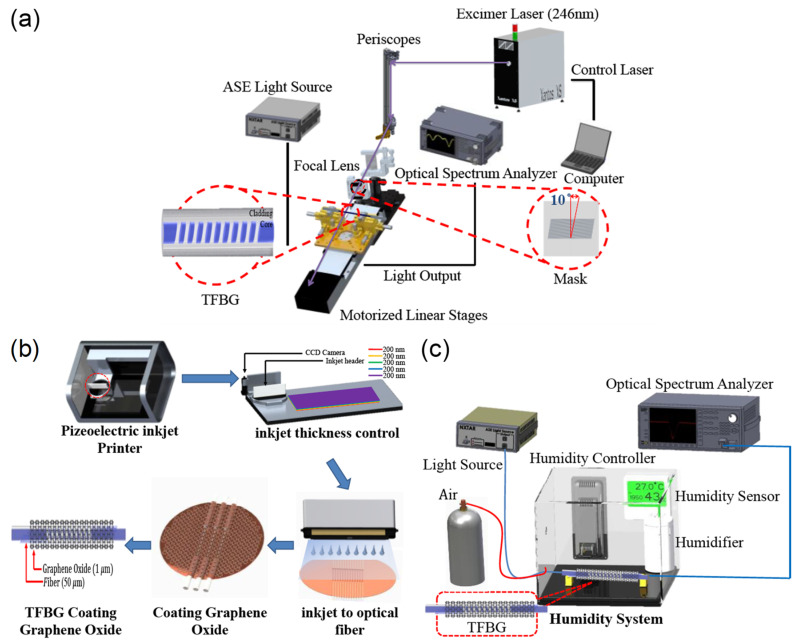
Point sensor. (**a**) Fabrication process of the sensing fiber. (**b**) Fabrication process of the graphene oxide coating (**c**) Experimental setup. Adapted with permission from ref. [128]. Copyright 2017 *MDPI*.

Yung-Da Chiu et al. [128] (Figure 26) investigated the impact of etched diameter on tilted FBGs coated with GO, with a range between 20 and 60 µm. A response time of 12.25 min was demonstrated. It was found that the 20 µm diameter variant offered the highest sensitivity, at 10 pm/%RH between 20%RH and 80%RH. The dynamic range is 20%RH–80%RH. The etching process was facilitated by using buffered oxide etch. A piezoelectric inkjet printer was used to spray an aqueous solution of GO onto the surface of the optical fiber, then allowed to heat and cool for stronger bonding. The response of the coating to increasing RH is a change in refractive index, which affects the cladding mode and thus the interference at recombination. The resulting resonant wavelength shift is blue shifts with rising RH. Z. Harith et al. [129] used a tapered POF coated with seeded Al-doped ZnO for measuring RH. The sensitivity is 0.0386 mV/%RH between 50%RH and 75%RH, and the dynamic range is 50%RH–75%RH. The cladding of the POF was removed as part of the tapering process via chemical etching. Then, a ZnO nanocrystal seed layer was prepared by the sol-gel method and dip-coated onto the tapered fiber. Increasing the local RH leads to water adsorption on the fiber surface, which increases the effective refractive index of the coating and strengthens the leakage of light. The transmission at the output of the fiber corresponds to the RH.

**Figure 27:**
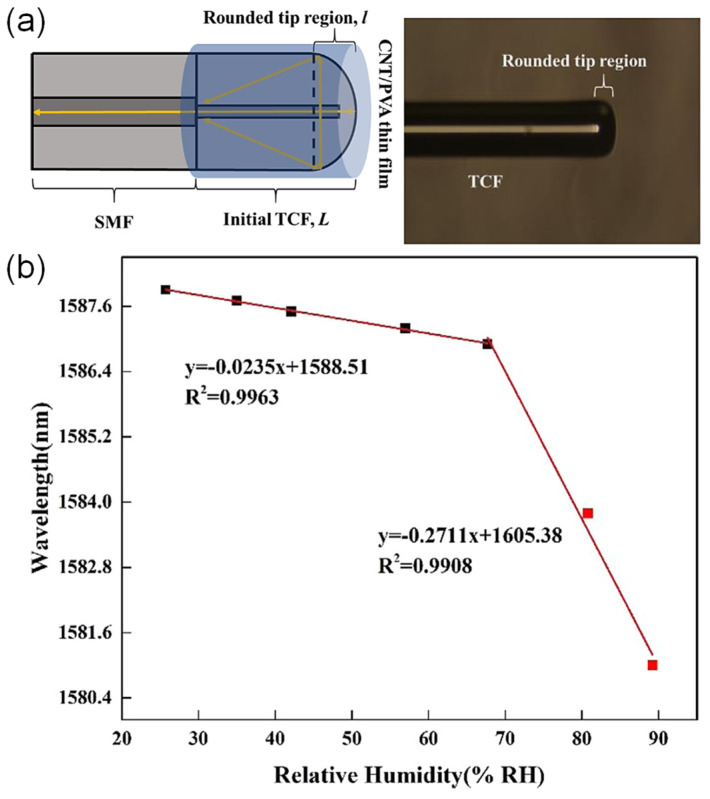
Point sensor. (**a**) Schematic of the sensor head and micrograph of the sensor head under 10x magnification. (**b**) Relationship between RH and wavelength. Adapted with permission from ref. [131]. Copyright 2018 *Elsevier*.

A cascaded peanut-shaped structure coated with PVA was proposed by Zhaowei Wang et al. [130]. The aim of the unusually shaped MZI was to use abrupt changes in geometry to partially convert the core mode into cladding modes. The sensitivity is 553.36 pm/%RH between 47%RH and 72%RH, while the dynamic range is 27%RH–72%RH. The peanut-shaped structure was created by cutting strands of fiber and splicing them to fuse into a sphere. Pairs of spheres are then fused together. Dip coating was used to coat the fiber with PVA. The interference conditions vary depending on the refractive index of the coating, which is dependent on the ambient RH. The interference spectrum was monitored for wavelength shifts as a function of RH. Q. F. Ma et al. [131] (Figure 27) combined carbon nanotubes and PVA for a composite coating on a thin-core fiber tip for RH measurement. The response time is 1 s. The sensitivity is 0.4573 dB/%RH between 70%RH and 87%RH, and the dynamic range is 30%RH–87%RH. A section of thin-core fiber was spliced onto the SMF, then reshaped using the same fusion splicer. This forms an inline Michelson interferometer. The tip was dip-coated with a blend of carbon nanotubes and PVA. The fundamental mode propagating through the SMF excites additional cladding modes at the interface with the thinned core, which reflects off the curved end (i.e., coating and air interfaces) and coherently recombine at the SMF. Changes in the local RH affect the refractive index of the coating, which changes the phase of the cladding modes, resulting in a change in the interference pattern and thus wavelength shift.

Bo Wang et al. [132] constructed an FPI at the end of an optical fiber using a layer of agar. The LOD is 0.6%RH, and the response time is 340 ms. The sensitivity is 4.20 nm/%RH between 25%RH and 95%RH, and the dynamic range is 25%RH–95%RH. A section of SMF was cleaved and dipped into a hot agar solution. The resulting thin film is leveraged as a resonant etalon whose optical path length changes with the absorption of moisture in its coating. Consequently, a shift in the interference spectrum can be observed. Bobo Gu et al. [133] developed a thin-core fiber modal interferometer for humidity sensing, relying on layer-by-layer self-assembly of a polycation, poly (4-vinylpyridinium chloride), and polyanion, sodium alginate. The response time is 15 s. The sensitivity is 100 pm/%RH between 40%RH and 95%RH, and the dynamic range is 40%RH–95%RH. The ISAM method was used for the layer-by-layer assembly on the surface of a thin-core fiber after it was spliced to two ends of SMF. Core modes are partially excited to cladding modes at the junctions of the fiber splices and interfere at the second junction. Changes to the refractive index of the coating modulate the differential phase between the modes, leading to shifts in the interference spectrum as a function of RH.

An HCF with a polyimide film was used as a humidity sensor by Ce Bian et al. [134]. The response time is 4 s. The sensitivity is 1.309 nm/%RH between 40%RH and 80%RH, and the dynamic range is 40%RH–80%RH. A film of polyimide was attached to a section of HCF spliced to SMF by soaking and pulling. By controlling the soaking time and the pulling speed, a thickness coating can be made. The resulting structure is a series of cascaded Fabry-Perot etalons, whose film thickness and thus cavity length varied with RH. However, this design resembling a diaphragm is likely to be susceptible to acoustic and vibration noise. Cheng Li et al. [135] reported a miniature fiber-tip Fabry-Perot-based humidity sensor using a GO diaphragm. The LOD is 0.4%RH, and the response time is 60 ms. The sensitivity is 82 pm/%RH between 10%RH and 70%RH and 630 pm/%RH between 70%RH and 90%RH. The dynamic range is 10%RH–90%RH. A section of silica capillary was spliced to SMF. A GO solution was synthesized by a modified Hummers method. A fiber tip with an open-air cavity was dipped into the solution until it touched the GO dispersed layered flake and left to dry to form the diaphragm. The GO layer serves as a mirror, whose thickness varies with different RH to change the cavity length. This resulted in changing interference conditions and a shift in resonant wavelength.

Hamid E Limodehi et al. [136] created a multi-channel fiber-optic dew and humidity sensor based on SPR. The LOD is 5%RH, and the dynamic range is 10–80%RH. For each sensing fiber, a section of MMF is side-polished and coated with gold. Surface plasmons resonance occurs on the gold surface when the conditions for phase matching are met. This condition can be triggered by adsorbed moisture on the fiber surface. Since surface plasmons are lossy waves, the transmission is a function of the RH. Jing Chai et al. [137] showed that a coupling agent can improve the sensitivity of FBG-based humidity sensors. The new FBG sensors are reported to be better performing than those without the coupling agent. The response time varies between 70 and 110 s. The sensitivity range is between 3.38 and 29.35 pm/%RH between 25%RH and 90%RH. The dynamic range is 25%RH–90%RH. A pretreatment of FBGs improves the bonding strength between a polyimide film and the FBG section. To maximize the stress transfer of the polyimide to the fiber, the coupling agent N-hydroxyethyl ethylenediamine can combine with the hydroxyl group of the FBG cladding and the amino group of the polyimide.

**Figure 28:**
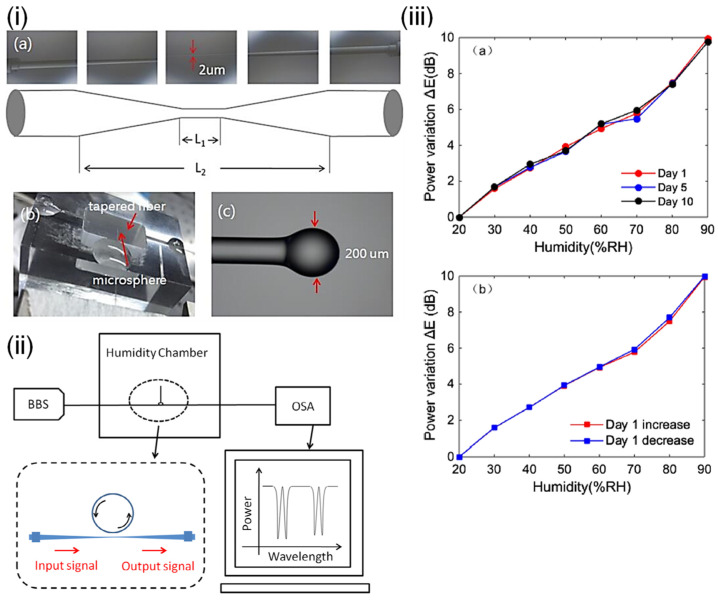
Point sensor. (**i**) (**a**) Schematic of tapered fiber. (**b**) Photograph of the packaged sensor. (**c**) Microscope image of the microsphere. (**ii**) Experimental setup. (**iii**) (**a**) RH vs. power variation over time. (**b**) Hysteresis graph. Adapted with permission from ref. [139]. Copyright 2018 *Elsevier*.

Kasun Prabuddha Wasantha Dissanayake et al. [138] presented another graphene-oxide-based LPFG for RH measurements. The sensitivity is 0.15 dB/%RH between 60%RH and 95%RH, and the dynamic range is 20%RH–95%RH. An improved Hummer’s method was employed to synthesize the GO dispersion, which was used to functionalize a section of LPFG via a dip-coating technique. The functional layer absorbs water molecules and changes its refractive index. Although the interference spectrum shifted as a result of RH variation, the intensity modulation was more significant and thus used to provide a measure of RH. Lei Liang et al. [139] (Figure 28) proposed and demonstrated a means to quantify RH using a microsphere-based whispering gallery mode resonator. The response time is 10 min. The sensitivity is 0.11 dB/%RH between 20% and 70% and 0.21 dB/%RH between 70% and 90%. The dynamic range is 20%RH–90%RH. With increasing RH, the accumulation of moisture on the tapered fiber changes the effective refractive index and thus coupling efficiency to the microsphere. The effect becomes increasingly pronounced at high RH, and thus the sensitivity is higher.

Ag-decorated ZnO nanorods have been exploited by Shweta Jagtap et al. [140] with a focus on sensitivity and dynamic range. The response time is 10 s, and the dynamic range is 20%RH–95%RH. PVA was used as a temporary binder for the composite paste comprising ZnO and Ag-ZnO. A section of cladding-removed POF was coated with Ag-ZnO paste and UV-dried. The sensing mechanism involves the adsorption of water molecules on the functional coating. The surface reaction changes the refractive index of the coating and affects the optical confinement of light, leading to transmission losses as a function of RH. Tingting Yuan et al. [141] leveraged an array of optical PMMA microfibers for humidity sensing. The response time is 30 s, and the sensitivity is 0.57%/%RH between 35%RH and 85%RH. The dynamic range is 35%RH–85%RH. The PMMA microfibers served as a bridge between two indium tin oxide (ITO) glasses, connected to the light source and detector via POFs. The array of microfibers facilitates a large surface area for water interactions, which lend themselves to increasing optical leakage with increasing RH. The transmission can be monitored to provide a readout of the RH.

Poonam D. Mahapure et al. [142] studied transmission-type humidity sensors using poly(dimethyl siloxane), poly(methyl methacrylate), PVA, and poly(N-vinylpyridine) (PVP) as substrates, and silver nanoparticle (nAg)/0.1% PVP (S1) and nAg/0.1% PVA (S2) as the sensing elements. A range of sensitivities from 0.84 to 0.92(/%RH) between 6%RH and 94%RH was reported. The dynamic range is 6%RH–94%RH, and the response time is 6 s. Yanhua Zhao et al. [143] investigated a Fabry-Perot-based humidity sensor using a polyimide membrane. The response time is 2 min, and the sensitivity is 0.164 nm/%RH between 20%RH and 95%RH. The dynamic range is 20%RH–95%RH. To form the Fabry-Perot etalon section of MMF was glued to a ceramic ferrule and then a cover glass with the polyimide film. The film was annealed to form a porous surface for increased interaction area with water molecules. With the absorption of water vapor, the functional coating swells and changes the optical path length, leading to shifts in the interference spectrum.

Yu Shao et al. [144] tested an SPR sensor based on a side-polished SMF with a gold coating followed by a PVA coating. By tracking the resonant wavelength shift with changing RH, a relationship was established. The response time is 251 ms, and the monotonic sensitivity is 1.01 nm/%RH between 40%RH and 90%RH. The dynamic range is 40%RH–90%RH. George Y. Chen et al. [145] demonstrated a humidity sensor using light-sheet excitation of skew rays for probing an MMF coated with PDDA/PSS. The LOD is 0.099%RH or 0.007%RH. The response time is 115 ms. The sensitivity is 0.04 dB/%RH between 10%RH and 60%RH and 80%RH–94%RH, and 0.57 dB/%RH between 60%RH and 80%RH. The dynamic range is 10%RH–94%RH. The polyelectrolyte coating was applied to the MMF surface as a binder via the ISAM method. Gold nanoparticles were then attached via electrostatic forces. A series of lenses converted a beam of light into a light sheet before it translated into a precise group of skew rays. The skew rays enable a far higher number of reflections per unit length of fiber, which increases its interaction strength with the functional coating. The gold nanospheres give rise to LSPR for further enhancement. With increasing RH, the water content in the coating attenuates the much enhanced evanescent field of light, thus changing the transmission.

**Figure 29:**
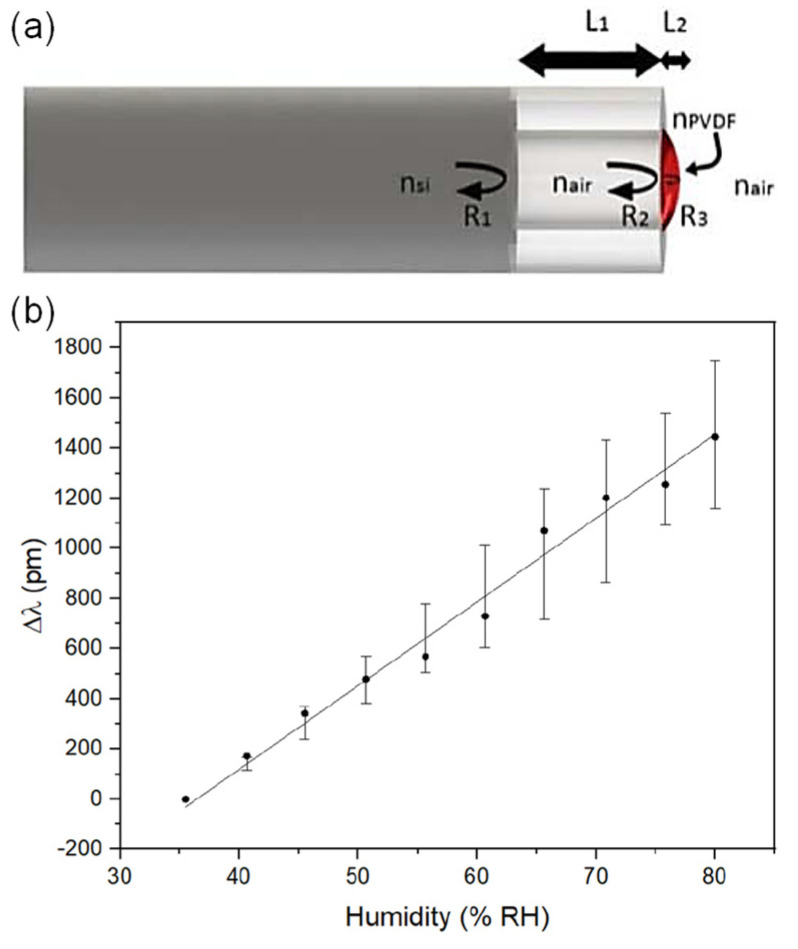
Point sensor. (**a**) Conceptual design of sensor head. (**b**) Wavelength shift with different RH. Adapted with permission from ref. [147]. Copyright 2019 *IEEE*.

A two-mode microfiber knot resonator coated with reduced GO was designed and fabricated by Le [146]. The LOD is 0.99%RH, and the response time is 7.2 s. The sensitivity is 603 pm/%RH between 35%RH and 90%RH, and the dynamic range is 35%RH–90%RH. Reduced GO was first prepared via ultra-sonification and hydrothermal reaction. The flame-tapering technique was applied to taper the optical microfiber from SMF. Then, a freestanding end of the microfiber was tied into a knot. The microfiber knot was spin-coated with the reduced GO and dried. Owing to the absorption of water molecules, the effective refractive index seen by the guided modes changes with varying RH, which affects the optical path length and thus the resonant wavelength. António Vaz et al. [147] (Figure 29) used a polyvinylidene fluoride FPI for RH measurements. The sensitivity is 32.54 pm/%RH between 35%RH and 80%RH, and the dynamic range is 35%RH–80%RH. The sensing etalon was fabricated by splicing a section of hollow silica tube to SMF. Then, the fiber was coated with a PVDF thin film that served as a mirror. The interferometer experiences a shift in its interference spectrum due to the change in the refractive index of the functional coating as a function of RH.

Chunhua Tan et al. [148] explored the combination of a lithium chloride-polyvinyl alcohol film coated on an optical fiber, using a two-channel Fresnel reflection method. The LOD was reported to be 1%RH, and the response time is 100 s. The dynamic range is 22%RH–83%RH. Lithium chloride was dissolved in deionized water, and a section of an optical fiber was dip-coated with the solution. The reflectivity of the sensing fiber tip varies with RH, as it influences the refractive index of the functional coating. A reference fiber provided the reference power to compensate for fluctuations in the intensity of the laser source. Erwin Maciak et al. [149] employed a low-coherence interferometer, otherwise known as a white-light interferometer, for humidity monitoring. A Fabry-Perot etalon coated with commercially available Nafion was used as the sensing section. The response time is 5 s, over a dynamic range of 5.5%RH–80%RH. The probe was constructed from graded-index MMF. The functional coating was attached to the end-face via successive dip coating. The Fabry-Perot etalon formed by the Nafion coating changes its optical path length with the onset of water absorption, thus affecting the resonant wavelength.

Another Fabry-Perot-based humidity sensor was developed by Jinze Li et al. [150]. It involves using an HCF and a hydroxypropyl methylcellulose hydrogel film. The response time is 2.25 s, and the dynamic range is 40–99%RH. A section of HCF was spliced to SMF. A hydroxypropyl methylcellulose film was coated on the end-face of the HCF to form an FPI. Like previous similar designs, the interference spectrum shifts with increasing optical path length from increasing RH. Ke Xu et al. [151] explored a coatingless microfiber knot resonator, taking advantage of the strong water absorption near 1960 nm. The response time is 1.13 or 0.8 s, depending on the direction of RH change. The sensitivity is 0.18 mW/%RH between 70%RH and 90%RH, or 0.034 dB/%RH or 10 pm/%RH between 35%RH and 95%RH. The dynamic range is between 28%RH and 95%RH. The fabrication of the sensor head started with an SMF, which was tapered using the flame-brushing technique before it was tied into a knot. The knot was tight enough to ensure self-coupling between the knot and the adjacent microfibers for enabling resonance. The coatingless microfiber knot resonator does not need a functional coating to become sensitive to RH. Its evanescent field allows light to be absorbed by the water molecules in the local environment, providing a measure of the RH.

Mahboubeh Dehghani Sanij et al. [152] explored the use of tetrakis (4-sulonatophenyl) porphyrin (TPPS) thin film on optical fibers for humidity sensing. The demonstrated LOD is 1%RH, and the response time is 2 min. The LOD is 1%RH. The sensitivity is 9.4 pm/%RH between 1% and 85%, and the dynamic range is RH0%RH–99%RH. A multimode fiber was dipped in a diluted solution of the tetrabutylammonium salt of TPPS in CH_2_Cl_2_ with ultraviolet laser irradiation. The irradiation power was optimized to produce the best morphological results on the TPPS thin film attached to the fiber end-face. Reflection spectroscopy was applied to analyze the local environment. Both the refractive index and absorption coefficient depend on the environmental humidity. The thin film also exhibits low-quality factor random Fabry-Pérot interference patterns. Miguel Hernaez et al. [153] reported a nanostructured polyethylenimine and GO coating with LMR. The response time is 50 ms, and the dynamic range is 20%RH–90%RH. The sensing fiber was prepared from a section of cladding-removed multimode fiber. The surface was first sputtered with SnO_2_ film to facilitate LMR absorption bands. Poly(ethyleneimine) (PEI) solution in deionized water and GO powder were used for the layer-by-layer assembly of the sensitive layer on top of the SnO_2_ underlayer. The refractive index of the PEI/GO coating is sensitive to changes in RH, which results in wavelength shifts of the LMR band.

**Figure 30:**
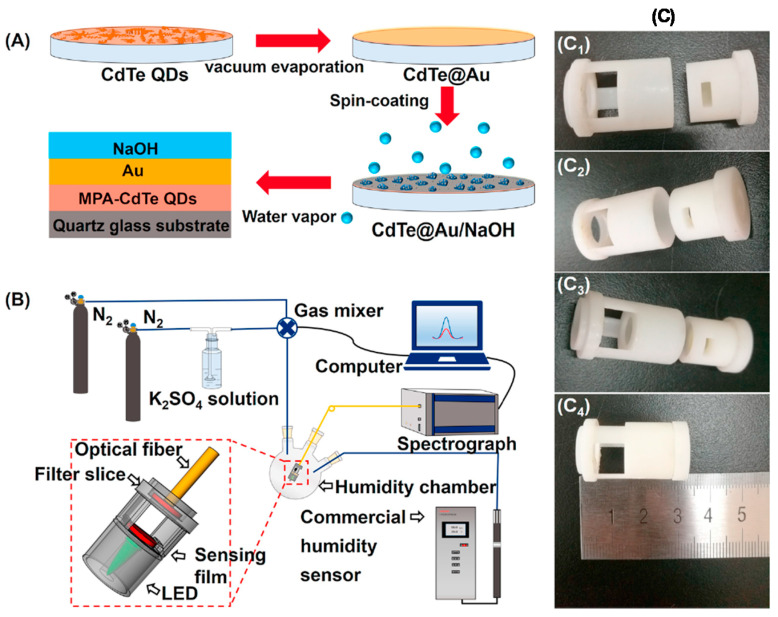
Point sensor. (**A**) Fabrication process of the sensing film. (**B**) Experimental setup. (**C**) Photographs of the device. Reprinted with permission from ref. [154]. Copyright 2019 *Elsevier*.

A nanocomposite film was presented by Mingyu Chen et al. [154] (Figure 30), which offers a highly stable operation. The response time is 30 s. The sensitivity is 254 a.u./%RH between 5%RH and 50%RH and 910 a.u./%RH between 50%RH and 97%RH. The dynamic range is 5%RH–97%RH. The nanocomposite film was obtained by depositing Au nanoparticles on the surface of 3-mercaptopropionic acid (MPA) capped CdTe quantum dots (QDs) and then modifying NaOH (CdTe@Au/NaOH). The CdTe@Au/NaOH film forms compound salts that can be dissolved or crystallized with changes in ambient RH, creating significant changes in the absorption of green light. The sensor head construction involves a light-emitting diode irradiating an end-face of an optical fiber through the sensing film. The sensing film modulates the transmitted power of light as a function of RH. Oskar Arrizabalaga [155] developed an FPI using an off-center polymer cap connected to an optical fiber facet. The LOD is 0.04%RH. The sensitivity is 148 pm/%RH between 10%RH and 95%RH, and the dynamic range is 10%RH–95%RH. The fabrication process begins with an SMF, which was then coated with UV-curable NOA 81 polymer. The endcap formed was intentionally misaligned to create two paths of reflected light for interference. Upon absorbing moisture, the polymer endcap swells and changes the differential path length in the inline interferometer and thus produces a shift in the interference spectrum.

Rang Chu et al. [156] reported an all-optical GO-based humidity sensor using a side-polished twin-core fiber Michelson interferometer. The response time is 3.6 s. The sensitivity is 2.72 nm/%RH between 40%RH and 75%RH. The dynamic range is 40%RH–75%RH. A twin-core fiber was side-polished such that one core was exposed to the outside environment. A GO solution was prepared by an improved Hummer’s method and was used to coat the exposed core. The detection system involves the use of a fiber taper to split light from SMF to the twin cores. A bent section of fiber served to impose an initial differential path length to create a starting set of interference fringes. The effective refractive index of the fundamental mode of the polished core is affected by the humidity-induced refractive index change of the GO. As a result, the optical path length difference between the two cores changes for different RHs, and result in a shift of the interference spectrum.

Aneez Syuhada et al. [157] fabricated a non-adiabatic tapered fiber coated with humidity-sensitive GO/PVA nanocomposite film and monitored the transmitted intensity count as a function of applied RH. A sensitivity of 0.0062 (a.u.)/%RH between 20%RH and 99.9%RH was demonstrated for the dynamic range of 20%RH–99.9%RH. Arnaldo G. Leal-Junior et al. [158] proposed the use of a smartphone’s light-emitting diode (LED) flashlight and camera for measuring humidity through a silica gel sphere that produces fluorescence according to the RH. POFs delivered and collected light. Proof-of-concept data were presented using standard lab equipment in place of a smartphone. A sensitivity of 0.026 (a.u.)/%RH between 25%RH and 80%RH was shown, with a dynamic range of 25%RH–80%RH.

Deyhaa A. Resen et al. [159] proposed and demonstrated a heterodyne method for humidity measurement, using an FBG to filter and shift the center wavelength of one path of light relative to the other. A sensitivity of 0.142 GHz/%RH between 23.8%RH and 83.4%RH was reported, with a dynamic range of 23.8%RH–83.4%RH. Marcin Procek et al. [160] fabricated silk fibroin thin films using the spin-coating method. The optical path length was characterized as a function of RH based on spectral observation. The response time is 7.1 s, and the dynamic range is 7%RH–90%RH.

Humidity-dependent plasmonic coupling within a 2D network of gold nanoparticles was exploited by Marco A. Squillaci et al. [161]. The result was an all-covalent network of gold nanoparticles connected by di-thiolated polyethylene glycol molecules. A shift in the resonance wavelength is governed by the humidity-induced changes in the average interparticle separation. The response time was reported to be 200 ms. Md Ashadi Md Johari et al. [162] sensitized microbubble resonators with PVA or poly methyl meth acrylate (PMMA) coatings to absorb moisture and change the effective index seen by the resonant modes. A 2 μm diameter optical microfiber was used to couple light in and out of the microresonator. By monitoring the resonant wavelength, a response time of 180 s was observed. The sensitivities are 0.1311 dB/%RH (PVA-coated), 0.0462 dB/%RH (no coating) and 0.0329 dB/%RH (PMMA-coated) between 40%RH and 95%RH. The dynamic range is 40%RH–95%RH.

N. Kaur Sidhu et al. [163] carried out numerical simulations of polyimide-coated fibers that contain a π-phase-shifted FBG. The mechanism of moisture absorption was modeled and simulated, which includes transport of diluted species physics to calculate the variation of moisture concentration in the hygroscopic coating. The concentration of water was scientifically estimated according to time-dependent isotropic diffusion, based on Fick’s law. The simulation results predict the distribution of the stress in polymer with different coating thicknesses. Experimental verification was conducted, and the response time was 10 min. The sensitivity is 1.11 pm/%RH between 20%RH and 50%RH, and the dynamic range is 20%RH–50%RH. Rozalina Zakaria et al. [164] demonstrated SPR in a titanium-coated side-polished optical fiber that is protected by a layer of 2D molybdenum disulfide (MoS_2_) or tungsten disulfide (WS_2_). Only the Ti/MoS_2_ showed a response when changing the RH from 58% to 88%. Hence, MoS_2_ exhibits higher sensitivity on Ti with 36-nm thickness compared with that of WS_2_.

**Figure 31:**
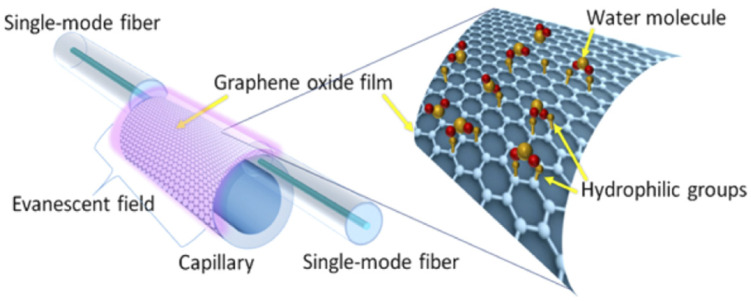
Point sensor. Humidity sensing based on GO and evanescent tunneling. Adapted with permission from ref. [166]. Copyright 2019 *OSA*.

Susana Novais et al. [165] dip-coated agarose gel onto a 30 mm-long section of coreless fiber spliced to SMF. Relying on multimode interference, the gel changes refractive index with RH, resulting in spectral shifts as a function of RH. A LOD of 1.1%RH was achieved. A response time of 30 min was reported, with a sensitivity of 44.2 pm/%RH between 60%RH and 98.5%RH. The dynamic range is 60%RH–98.5%RH. Yun Liu et al. [166] (Figure 31) coated a fiber-butt-coupled microcapillary with graphene oxide (GO) film. The sensing mechanism involves the interaction between the evanescent field on the capillary surface and water vapor molecules adsorbed on the GO layer. The thin-walled capillary enables the excitation of high-order modes. The GO layer also increases the refractive index of the external medium to strengthen the evanescent field. Both choices help to increase light-matter interaction and thus improve the sensitivity. A sensitivity of 0.057 nm/%RH was attained, and a dynamic range of 30%RH–70%RH was tested.

Anand M. Shrivastav et al. [167] constructed an FPI by depositing a chitosan polymer matrix within the holes of a 140 μm length suspended tri-core fiber spliced to SMF. Real-time breath monitoring was demonstrated as a potential application. The functional coating changes its refractive index with varying RH, which affects the effective optical path length of the etalon and thus the spectral response. The three cores provided an adequate surface area for the deposition of the chitosan material. The sensitivity is 81.05 pm/%RH between 90%RH and 95%RH, and the response time is 80 ms. The dynamic range is 70%RH–95%RH. Anuj K. Sharma et al. [168] explored 2D materials/heterostructure for SPR, with porous silica film-coated multimode fibers and angular light excitation (e.g., meridional and skew rays). It was found that the graphene variant exhibited the largest optical response. The LOD is 0.0041%RH, the sensitivity is 171.11 dB/%RH near 99%RH, and the dynamic range is 0%RH–100%RH.

Ce Bian et al. [169] published two designs based on single-mode multimode cascaded designs. The first used calcium alginate (CaAlg) hydrogel coated on a section of no-core fiber, sandwiched by two SMFs to form an FPI. Changes in the humidity-influenced refractive index of the functional coating affect the effective indices of the multimode cavity, which produces a corresponding spectral shift. The response time is 3 s. The sensitivity was measured to be 0.38 dB/%RH between 30%RH and 80%RH. The dynamic range is 30%RH–80%RH. Building on the same functional material and sensing mechanism, Ce Bian et al. [170] demonstrated a modal MZI using consecutive stages of SMF-MMF-SMF-MMF-SMF. The response time is 2 s, and the sensitivity is 0.48346 dB/%RH between 15%RH and 80%RH. The dynamic range is 15%RH–80%RH. Carmen E. Domínguez-Flores et al. [171] explored a dual-FPI design with SnO_2_ coating. Increasing RH decreases the RI of the SnO_2_ thin film, which reduces the reflectance of the fiber tip end-face and thus decreases the visibility of the interference fringes. The sensitivity is 39.49 × 10^−3^%/RH between 40%RH and 90%RH, and the dynamic range is 40%RH–90%RH.

**Figure 32:**
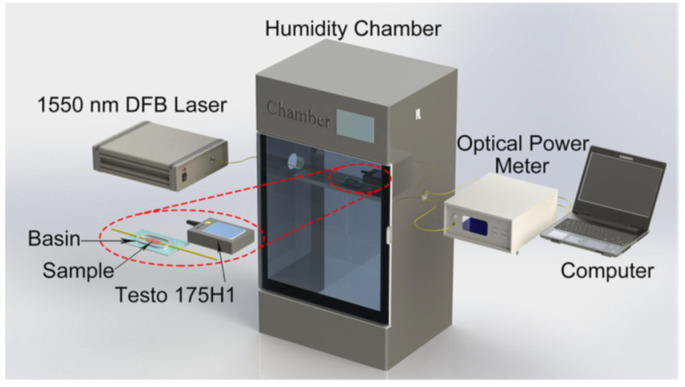
Point sensor. Schematic of relative power measurement of optical microfiber overlaid with niobium disulfide. Reprinted with permission from ref. [172]. Copyright 2020 *Springer*.

Niobium disulfide (NbS2) was explored by Enze Zhang et al. [172] (Figure 32) with the goal of combining transition metal dichalcogenides with optical microfibers. The relative optical power was recorded as a function of wavelength and RH, and the resulting sensitivities are similar. The transmitted power is proportional to the RH. A sensitivity of 1.05 dB/%RH was demonstrated between 72%RH and 97%RH. The response time was reported to be 15 ms (it should be noted that the stabilization time is only a few ms), and the dynamic range is 72%RH–97%RH. Farah H. Hamza et al. [173] exploited sodium silica that changes its transmission window with varying RH, using a single mode and separately a multimode fiber. The sensitivities are 0.045 nm/%RH (SMFS) and 0.036 nm/%RH (MMFS) between 30%RH and 80%RH. The dynamic range is 30%RH–80%RH.

Hsin-Yi Wen et al. [174] used a U-shaped probe and electrospun functional nanofibers for humidity measurements. A polymer solution of poly(3,4-ethylenedioxythiophene) polystyrene sulfonate (PEDOT:PSS) doped in polyvinyl alcohol (PVA) was used. The sensor head is comprised of a chemically etched optical fiber bent in a U-shape, such that modal interference occurs. Coating-induced shifts in the interference spectrum led to a sensitivity of −0.990 nm/%RH between 20%RH and 80%RH. The dynamic range is 20%RH–80%RH. Exploiting the hydrophilic and humidity-sensitive characteristics of graphene oxide, Huo Shihang et al. [175] created an inline humidity sensors using a graphene oxide thin film on a side-polished section of optical fiber. Through water adsorption or release depending on the local environment, the phase delay of the cladding mode changes and influences the resulting interference. The reported sensitivity is 0.165 dB/%RH between 35%RH and 65%RH, and the dynamic range is 35%RH–65%RH.

**Figure 33:**
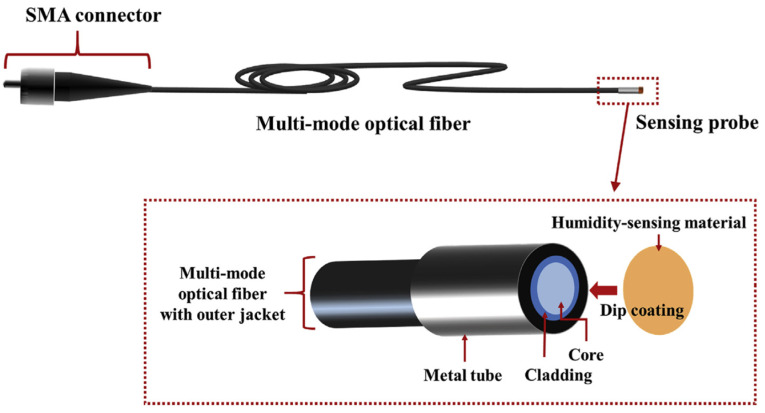
Point sensor. Humidity-sensing probe and the internal structure of the sensor head. Reprinted with permission from ref. [176]. Copyright 2020 *Elsevier*.

Hye Jin Kim et al. [176] (Figure 33) used PVDF in dimethyl sulfoxide (DMSO) and hydroxyethyl cellulose (HEC) and dip-coated the hydrogel mixture onto the end-face of a step-index MMF. The reflectance spectrum changes as a function of RH, of which different ratios of the mixed components resulted in different sensitivities (arbitrary units) when observing reflected power at a wavelength of 660 nm. A response time of 15 s was reported, and a dynamic range of 40%RH–95%RH. Hyong Su Yu et al. [177] explored the use of humidity-induced refraction change and an electrical position sensor to measure RH. Free-space light passes through an acrylic resin film, where the humidity-dependent RI changes the lateral position of light output and thus the position on the position sensor. The produced electrical current varies according to the position of the incident light. The sensitivity is 6.52–7.34 μA/%RH between 10%RH and 90%RH. The response time is 5 s, and the dynamic range is 10%RH–90%RH.

Jinze Li et al. [178] developed a carboxymethyl cellulose (CMC)/carbon nanotubes (CNTs) composite film. They constructed a humidity sensor by using an FBG connected to a hollow-core fiber (HCF), with a CMC/CNTs hydrogel composite film coated on the end-face of the HCF. Through experimental verification, the authors concluded that carbon nanotubes can increase the sensitivity of the hydrogel film. The measured sensitivity is 230.95 pm/%RH between 35%RH and 85%RH, and the dynamic range is 35%RH–85%RH. However, it was not clear what is the main sensing mechanism that contributes to a red shift of the resonant wavelength with increasing RH. Li Jia-xin et al. [179] designed a modal MZI using two sections of a spherical structure fabricated using arc discharge. The functional material used for the thin film is unknown. The sensitivity was reported to be 0.148 nm/%RH between 30%RH and 70%RH, and the dynamic range is 30%RH–70%RH.

Lin Zhao et al. [180] developed a quasi-distributed humidity sensor using FBGs coated with polyimide. Relying on the humidity-induced strain that transfers to the optical fiber, the authors demonstrated a LOD of 2%RH and sensitivity is 5.8 pm/%RH between 5%RH and 95%RH. The response time is 4.3 min, and the dynamic range is 5%RH–95%RH. Meng-Chang Hsieh et al. [181] approached humidity sensing with a phase-enhancement total-internal-reflection (TIR) heterodyne interferometer. s- and p-polarized beams can amplify the phase delay prior to heterodyne demodulation. A humidity-influenced PVA film serves as the transducer of the refractive index at the point of TIR. The reported LOD is 0.5%RH, and the sensitivity is 0.49°/%RH between 30%RH and 75%RH. The response time is 65 s, and the dynamic range is 30%RH–75%RH.

**Figure 34:**
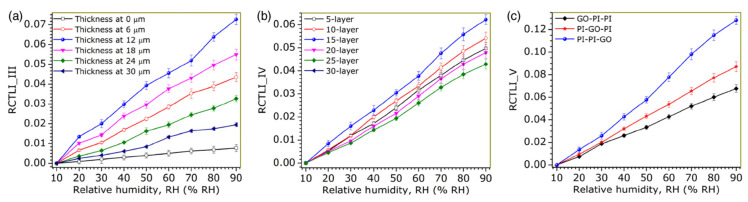
Point sensor. Effects of the (**a**) PI coating thickness, (**b**) number of GO coating layers, and (**c**) alternate PI–GO coating sequence on the sensitivities of the sensors at 25 °C (sampling time: 10 s). Reprinted with permission from ref. [183]. Copyright 2020 *OSA*.

Muhammad Quisar Lokman et al. [182] investigated nickel-doped zinc thin oxide (NZTO) perovskite as a suitable functional material for humidity sensing. The adsorption of water molecules on the surface of the thin film changes the reflectivity of light and the optical path length of the cavity, which provides a measure of RH. The sensitivity is 0.2657 nm/%RH between 60%RH and 90%RH, and the dynamic range is 60%RH–90%RH. Nainbing Zhong et al. [183] (Figure 34) developed a U-shaped plastic optical fiber evanescent-wave humidity sensor, using a variety of functional coatings, including graphene oxide and polyimide alternate deposition. The functional coating enhanced the attenuation of light in the coated section, leading to higher sensitivities. The highest sensitivity is 0.0017 (a.u.)/%RH between 10%RH and 90%RH, and the dynamic range is 10%RH–90%RH. The resulting sensor heads showed relatively low cross-sensitivities to temperature.

Nur Abdillah Siddiq et al. [184] exploited SnO_2_ on a planar optical waveguide to introduce humidity-dependent attenuation. A silicon wafer with a thermal oxide layer was used as the substrate and undercladding layer for the optical waveguide. Germanium and boron codoped silica layers were introduced into the substrate to form the core layer. The core layer was patterned using photolithography and etched using inductively coupled plasma (ICP) etching followed by spin coating of ZPU13 polymer to form the overcladding. The ZPU13 polymer coating was etched by using oxygen plasma down to the top surface of the waveguide core to enable evanescent wave interactions. The sensitivity is −2 dB/%RH between 56%RH and 90%RH, the response time is 2.5 s, and the dynamic range is 56%RH–90%RH. Pasquale Di Palma et al. [185] explored photonic crystal colloids auto-assembled on the tip of an MMF, which features a photonic bandgap modulation rather than an FPI modulation. Polystyrene (PS) nanoporous structures incorporated into a poly(N-isopropylacrylamide) (PNIPAM) hydrogel was used. The sensing mechanism involves a hydrogel shell that swells upon absorbing moisture from the air, which creates a wavelength shift as well as an amplitude variation in the photonic bandgap peak. The reported LOD is 1%RH. The sensitivity can be as high as 6000 pm/%RH at 95%RH. The dynamic range is 20%RH–95%RH.

**Figure 35:**
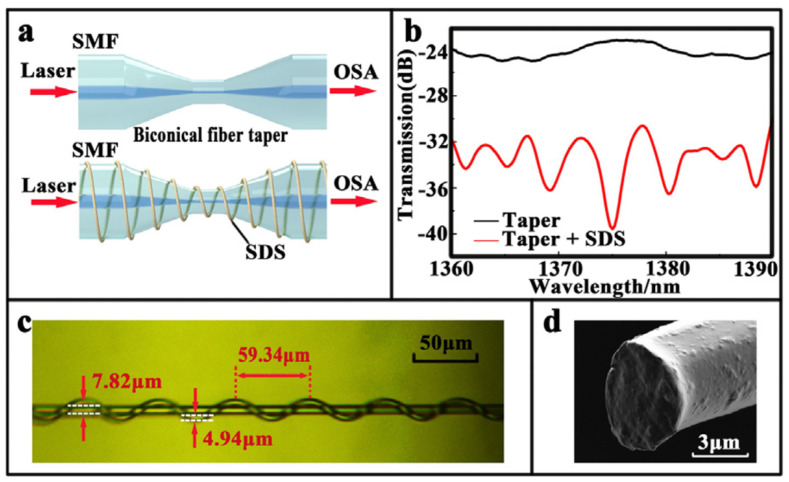
Point sensor. (**a**) Illustration of the biconical optical fiber taper and the spider-silk wrapped around the tapered fiber. (**b**) Interference spectra of the two versions. (**c**) Image of silked-coiled tapered fiber. (**d**) Scanning electron micrograph of spider dragline silk. Reprinted with permission from ref. [186]. Copyright 2020 *Elsevier*.

Zhihai Liu et al. [186] (Figure 35) investigated the use of spiker dragline silk to enhance the modal properties and sensitivity of an optical fiber taper by coiling the silk along the entire taper. It was found that the addition of silk increased the number of high-order modes, along with surface sensitization to humidity due to the silk absorbing moisture and changing its RI. The demonstrated response time is 374.46 ms. The sensitivity is 532 pm/%RH between 30%RH and 89%RH and 789 pm/%RH between 70%RH and 89%RH. The dynamic range is 30%RH–89%RH.

Catherine Grogan et al. [187] conducted humidity sensing with a free-space diffractive approach based on a holographically patterned thin photopolymer layer on a polymer (polydimethylsiloxane) (PDMS) layer, forming a bi-layer macro-cantilever that deflects light due to different responses of the layers to changing humidity. The LOD is 1.2%RH, and the sensitivity is 0.34%RH between 15%RH and 65%RH. The dynamic range is 15%RH–65%RH.

Cheng Zhou et al. [188] reported the use of a Vernier-enhanced cascaded FPI with a chitosan cavity for measuring RH. The modulation of the chitosan cavity within the cascaded cavities gives rise to a larger spectral shift. The response time is 987 ms, and the sensitivity is 7.15 nm/%RH between 40%RH and 92%RH. The dynamic range is 40%RH–92%RH. Chenyang He et al. [189] coated a fiber tip with poly(allylamine hydrochloride) (PAH) and silica nanoparticles, which serve as an FPI. For additional temperature sensing, thermochromic liquid crystal (TLC) was coated to replace the humidity-sensitive functional coating, and its reflectance spectrum was analyzed for temperature readings. The response time is 13.2 s, and the sensitivity is 0.43 nm/%RH between 55%RH and 90%RH. The dynamic range is 55%RH–90%RH.

Erfan Owji et al. [190] chemically etched SMF into an abrupt fiber taper, coated with 2D layers such as molybdenum disulfide (MoS_2_), molybdenum diselenide (MoSe_2_), and composition of graphene and graphene oxide (G/GO). The team analyzed the relative differentiation of attenuation as a function of RH. The conclusion was that G/GO was the most reliable coating among the three types. The tested dynamic range is 20%RH–90%RH. Husna Mardiyah Burhanuddin et al. [191] created a microcavity with an end-reflector made using ZnO embedded in PVA, which changes its RI with RH. A broadband laser source was used to interrogate the cavity and monitor its spectral shift in response to changes in RH. The sensitivity is 0.0743 nm/%RH between 60%RH and 90%RH, and the dynamic range is 60%RH–90%RH.

**Figure 36:**
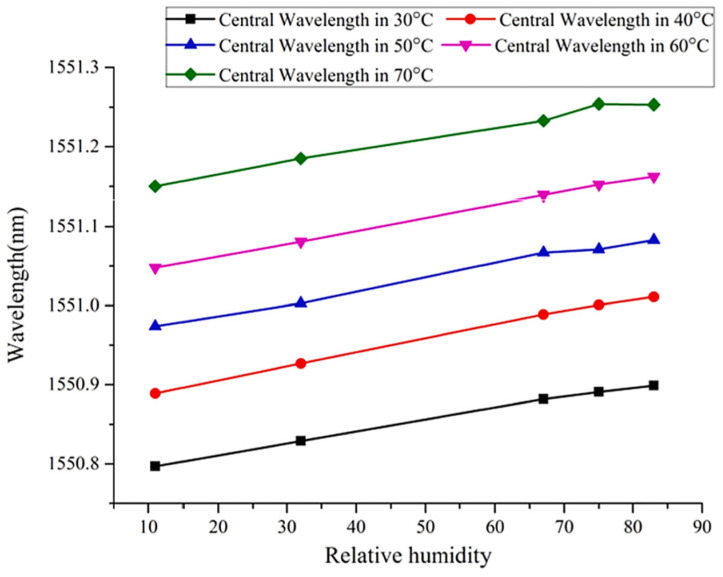
Point sensor. Bragg wavelength as a function of RH at different temperature settings. Reprinted with permission from ref. [193]. Copyright 2021 *Elsevier*.

Jia-Kai Wang et al. [192] explored the use of side-polished double D-shaped fiber for humidity sensing. On each side of the D-plane, a different combination of coatings was applied to support SPR for humidity and temperature measurements. On one side, copper film and ethanol film were deposited. On the other side, gold and PVA films were added. The aim was to construct a sensor head that is sensitive to both humidity and temperature through independent resonant wavelength shifts, though cross-sensitivity suppression and the range of testing are limited. The sensitivity to RH is 11.6 nm/%RH between 20%RH and 80%RH, and the dynamic range is 20%RH–80%RH. The sensitivity to temperature is 2.9 nm/°C in the range of 10–70 °C. Jianxin Zhang et al. [193] (Figure 36) also used polyimide-coated FPGs, to measure both RH and temperature. Instead of employing a reference FBG to isolate temperature data, the same FBG was used, and a calibrated temperature compensation algorithm was used to separate the variables. The resulting sensor can measure RH accurately at defined temperature levels that prevent signal ambiguity between RH readings at different temperatures. The sensitivity is 1.5 pm/%RH between 11%RH and 83%RH, and the dynamic range is 11%RH–83%RH.

For practical application, such as operation safety of transmission lines, Jie Zhang et al. [194] investigated the packaging method and installation fixture of separate FBGs for temperature and humidity sensing. Each FBG was coated with a different functional coating as the outmost layer targeting a specific measurand. The dynamic range is 40%RH–80%RH. Katerina Lazarova et al. [195] reported the use of amphiphilic poly(vinyl alcohol-co-vinyl acetal) copolymer, where a gold or palladium layer is deposited onto a flexible substrate such as polyethylene terephthalate, followed by spin coating polymer overlayer. The functional composite coating swells in response to increasing RH and changes the transmittance of light. The sensitivity is 0.02%/%RH between 5%RH and 75%RH and 0.18%/%RH between 75%RH and 95%RH. The dynamic range is 5%RH–95%RH.

**Figure 37:**
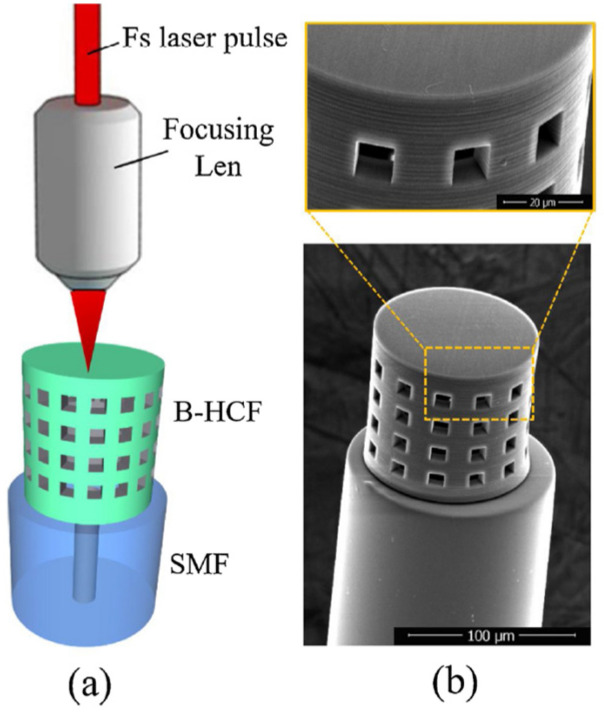
Point sensor. (**a**) Schematic diagram; and (**b**) scanning electron microscope image of the “castle style” FPI microcavity. Reprinted with permission from ref. [196]. Copyright 2021 *Elsevier*.

Mao-qing Chen et al. [196] (Figure 37) applied femtosecond laser inscription to the tip of an SMF to produce a 3D “castle style” FPI, which was then flushed and coated with PVA to form a humidity-sensitive reflective layer inside. The authors claim a larger contact area between water molecules and the humidity-sensitive materials, which was reported to result in high uniformity and faster response (not quantified in the paper). The sensitivity is 248.9 pm/%RH between 46%RH and 75%RH, the dynamic range is 46%RH–75%RH. Ning Wang et al. [197] used the water-absorbing property of graphene quantum dots. Two sections of flat cleaved SMF formed a cavity filled with graphene quantum dot solution. The RI and thus optical path length of the cavity depends on the local RH. The response time is 5.5 s. The sensitivity is 0.567 nm/%RH between 11%RH and 85%RH. The dynamic range is 11%RH–85%RH.

Sarah Kadhim Al-Hayali et al. [198] proposed and demonstrated a balloon-configuration fiber-optic probe involving bent SMF with polymer outlayer and gold nanoparticles coating. The sensing mechanism was reported to be modal interference, though it is also possible that LSPR contributed to the observed transmittance spectra. The spectral shift reveals the measured RH. The sensitivity is −0.571 nm/RH between 35%RH and 95%RH, and the dynamic range is 35%RH–95%RH.

Seyed Reza Hosseini Largani et al. [199] explored a set of bent optical fibers forming a U-shape, without a functional coating, to measure RH. The bent region allows the initial modes to couple to higher-order modes and back, forming a modal MZI. A double-pass configuration was set up with a Faraday rotator mirror to enhance the extent of mode coupling. The SMF version yielded the best results, though the sensing mechanism was not explained. The response time is 1 s. The sensitivity is 275 pm/%RH between 60%RH and 90%RH, and the dynamic range is 60%RH–90%RH.

**Figure 38:**
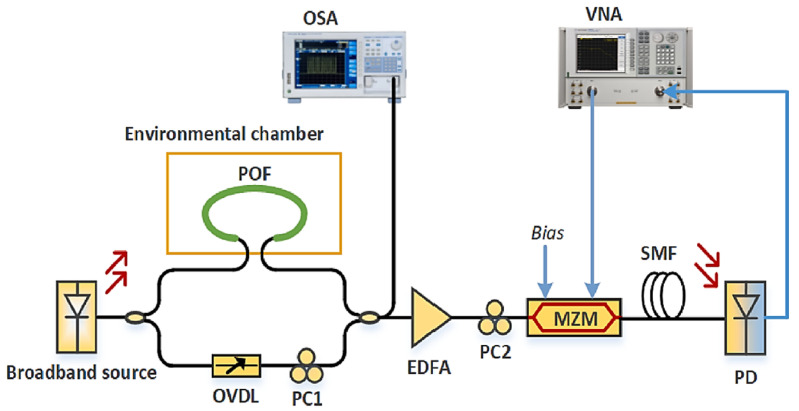
Point sensor. Experiment setup of the humidity sensor. (POF: polymer optical fiber; OVDL: optical variable delay line; PC: polarization controller; EDFA: erbium-doped fiber amplifier; MZM: Mach-Zehnder modulator; SMF: single-mode fiber; PD: photodetector; OSA: optical spectrum analyzer; VNA: vector network analyzer). Reprinted with permission from ref. [200]. Copyright 2021 *Elsevier*.

Xin Cheng et al. [200] (Figure 38) employed a single-passband microwave photonic filter in conjunction with a POF. The POF absorbs moisture from the air and swells up, changing its RI and thus optical path length. The resulting free spectral range of the broadband light passing through the POF incorporated in an MZI is changed. The spectral shift is translated into a frequency shift by the system. The LOD is 0.0119%RH, and the sensitivity is 84 MHz/%RH between 45%RH and 95%RH. The dynamic range is 45%RH–95%RH. Ying Wang et al. [201] employed SPR with a PVA film and gold coating on the flat surface of a side-polished POF. Redshift of the spectrum was observed with increasing RH due to changing RI of the PVA film. The response time is 0.44 s. The average sensitivity is 4.98 nm/%RH between 40%RH and 90%RH and can be as high as 10.15 nm/%RH. The dynamic range is 40%RH–90%RH.

**Figure 39:**
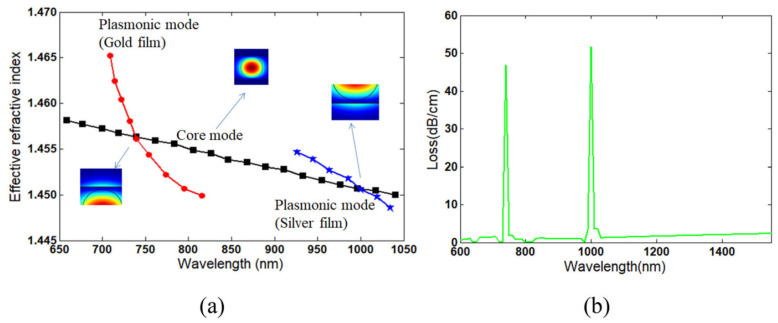
Point sensor. (**a**) Effective index as a function of wavelength, and (**b**) confinement loss spectra. Reprinted with permission from ref. [203]. Copyright 2021 *Taylor & Francis*.

Xixi Huang et al. [202] deposited a thin layer of silica cladding on an exposed-core fiber that had its coating and cladding mechanically removed. Then a layer of TiO_2_ was applied for humidity sensing. The sensing mechanism is evanescent wave attenuation. The response time is 25 s. The sensitivity is 5.35 µW/%RH between 15%RH and 50%RH and 1.94 µW/%RH between 50%RH and 95%RH. The dynamic range is 15%RH–95%RH. Yu Ying et al. [203] (Figure 39) also developed a double D-shaped fiber sensor, with toluene as thermally sensitive material and polyethylene as a humidity-sensitive material. Likewise, the dual-peak SPR analysis allows simultaneous measurement of RH and temperature. The sensitivity is 0.79 nm/%RH between 30%RH and 70%RH, and the dynamic range is 30%RH–70%RH.

Table 1 summarizes the smallest, and best LODs reported to date, and Table 2 ranks the shortest response times in the literature.

For comparison, some key electrical-based humidity sensors are summarized in Table 3. Although there is a lack of data on LODs for a fair comparison, the response times are generally shorter than those of optical humidity sensors. This could partly be attributed to the smaller size of the sensor head, which ensures a more instantaneous interaction with water molecules.

**Figure 40:**
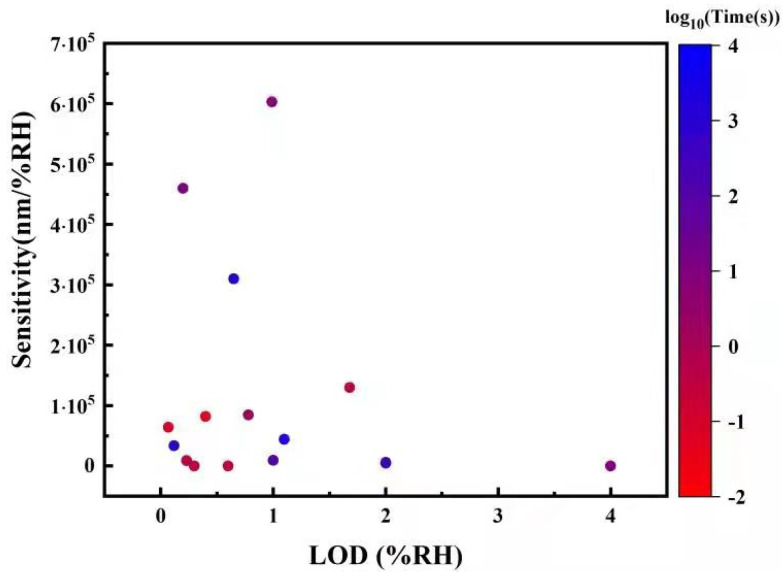
Relationship between limit of detection and sensitivity, with color representing log scale of time.

**Figure 41:**
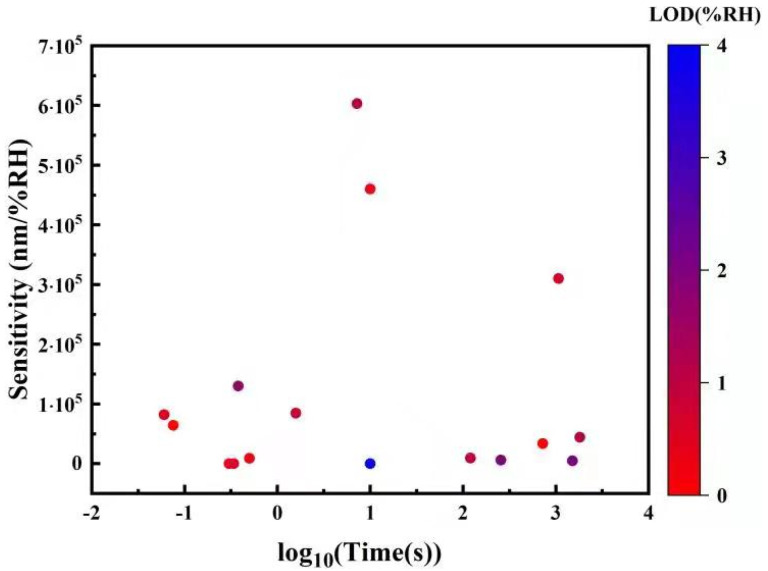
Relationship between log scale of time and sensitivity, with color representing limit of detection.

**Figure 42:**
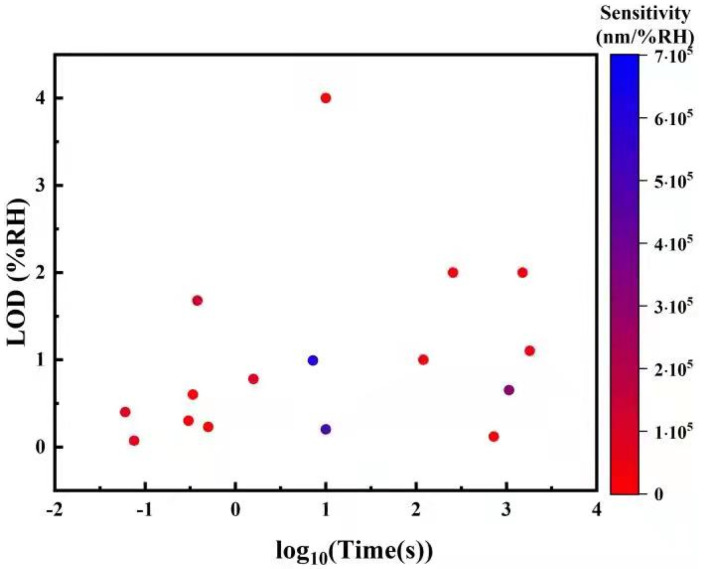
Relationship between log scale of time and limit of detection, with color representing sensitivity.

Among the point sensors and those with available data on the LOD, response time, and sensitivity, certain trends between the parameters have been observed. It can be seen in Figure 40 and Figure 41 that there is no particular correlation between the parameters. However, Figure 42 reveals a possible trend of lower sensitivities occurring at relatively medium response times and relatively low LODs. With more available data in the future, clearer correlations could be drawn.

### 3.4. Distributed Sensors

The ability to spatially resolve RH along a predetermined path can offer new insight into old problems. Places where distributed humidity sensors are highly sought after include pipelines, buildings, and plantation fields. A distributed fiber-optic humidity sensor can map the RH distribution and convey the information in real-time to a centralized system as a backbone of the internet of things (IOT) and big data for smart cities of the near future.

K. Ogawa et al. [219] were among the first to explore quasi-distributed humidity sensing. In 1988, they explored the concept of a microporous SiO_2_-cladded optical fiber. The LOD is, on average, ~5%RH between 20%RH and 95%RH. The response time is 45 s. The sensing range depends on the number of sensing points. The underlying mechanics involve water absorption by the porous cladding, which scatters light and attenuates the output power. W. C. Michie et al. [220] combined the functionalities of water and pH sensing with a distributed sensor using optical fiber and hydrogen. Being partly a water sensor, it can only distinguish between wet (100%RH) and dry rather than different RH levels. The spatial resolution is 1 m, while the distance range is 30 m. The sensing mechanism relies on micro-bending-induced optical loss by the swelling of hydrogen in response to water vapor absorption.

**Figure 43:**
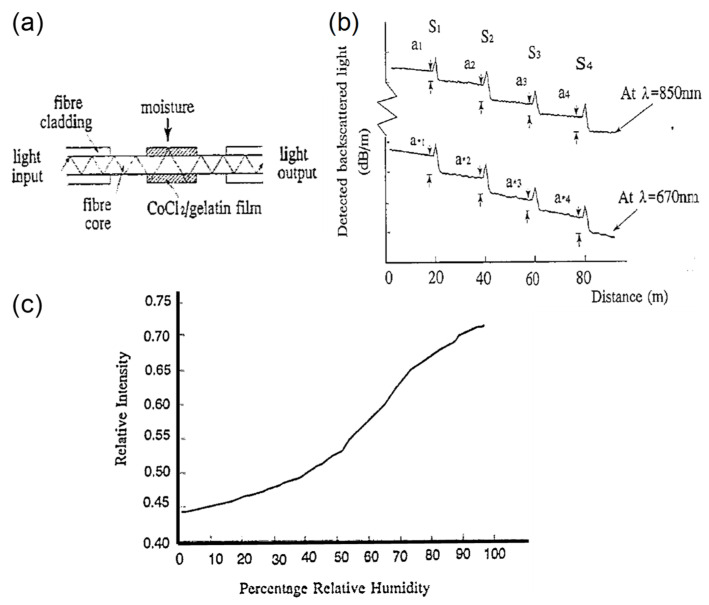
Distributed sensor. (**a**) Conceptual design of each sensing point. (**b**) Example OTDR trace with four sensing points. (**c**) Relative intensity as a function of RH. Adapted with permission from ref. [221]. Copyright 1995 *Elsevier*.

A. Kharaz et al. [221] (Figure 43) presented a distributed humidity sensor using periodically exposed-core coated with cobalt chloride. The LOD was reported to be 2%RH between 20%RH and 80%RH, and up to 80 m in distance range. A section of fiber cladding was replaced with cobalt chloride gelatin thin film. The working principle comprises a refractive index change upon moisture ingress, which provides a back-reflection proportional to the RH. Wei Bai et al. [222] reported a polyimide-coated FBG sensing network. The sensitivity is 1.639 pm/%RH between 24%RH and 83%RH, while the dynamic range is 24%RH–83%RH. Each FBG was dipped into a polyimide solution to obtain the desired coating thickness. The swelling of the coating strains the FBG, which changes its optical path length and shifts its Bragg wavelength.

A. Sascha Liehr et al. [223] were among the first to demonstrate a fully distributed humidity sensor, which is based on PMMA optical fiber. The achieved LOD is 1%RH between 30%RH and 90%RH. The response time is rather slow at 31 h due to the slow ingress rate of moisture through the PMMA fiber. The spatial resolution can vary from 10 cm to 2 m, up to a distance range of 93 m. The sensing mechanism is increased scattering of light with water absorption in the fiber, which can be detected and spatially resolved with optical time-domain reflectometry (OTDR). Peter J Thomas et al. [224] developed a fully distributed sensor for RH measurements based on the optical frequency-domain reflectometry (OFDR) interrogation of an optical fiber coated with polyimide. A LOD of 0.1%RH was demonstrated within the range of 15%RH–92%RH. The response time is 32 min. The spatial resolution is 1 cm, and the distance range is 62 m. The polyimide layer was deposited onto the optical fiber via dip coating. The sensor operates by allowing the swelling action of the polyimide to transfer strain to the fiber, which creates detectable variations in the local refractive index.

Peter J. Thomas et al. [225] also reported a high-spatial-resolution short-range distributed sensor for distributed RH measurements. The spatial resolution is only 2 mm between 15%RH and 80%RH. The distance range is 32 cm. The sensing mechanism is the same as the previous experiment. N. Zhou et al. [226] introduced a new concept of Au nanorod-coupled microfiber optical humidity sensor. It is a quasi-distributed sensor by design, and a microscope objective plus a translation system is required to analyze each nanorod. The sensor showed a LOD of 0.16%RH between 18%RH and 85%RH. The spatial resolution can be a small as 2 μm, limited by the crosstalk between neighboring cavities. The sensor operates by monitoring resonance detuning from the coupled WGM and LSPR modes of the hybrid structure.

**Figure 44:**
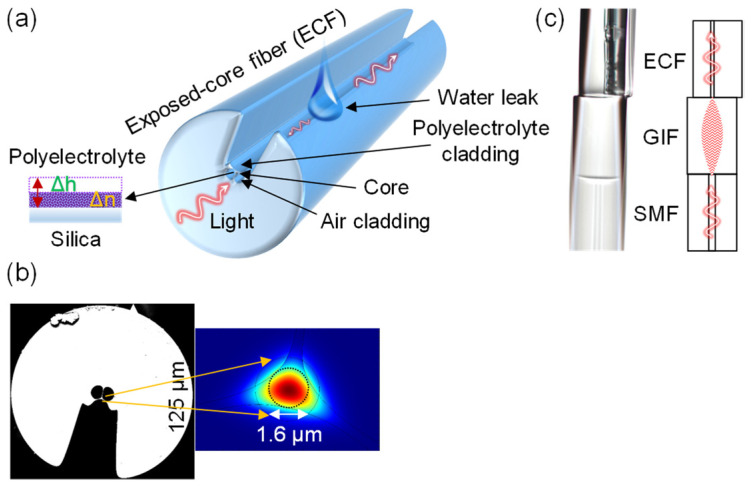
Distributed sensor. (**a**) Conceptual design of the sensing fiber. Inset: cross-section view of the polyelectrolyte coating. (**b**) Left: Scanning electron microscope image of the exposed-core fiber cross-section. Right: simulated mode profile in the core. (**c**) Splice sequence of the sensing fiber with an exposed-core fiber (ECF), a graded-index fiber (GIF), and a single-mode fiber (SMF). Reprinted with permission from ref. [227]. Copyright 2019 *IEEE*.

George Y. Chen et al. [227] (Figure 44) created short-range fully distributed humidity sensors based on OFDR and polyelectrolyte coating (PDDA/PSS). The LOD grows with distance along the fiber, from 0.3%RH to 10%RH, between 26%RH and 95%RH. The response time is 4 s. The spatial resolution is 4.6 mm, while the distance range is 38 cm. The PDDA/PSS was assembled on the exposed-core fiber using alternate dip coating. The sensing mechanism is the absorption of water, leading to changes in refractive index and back-reflected power along the fiber.

Humidity-induced strain measurement was investigated by Pavol Stajanca et al. [228] using polyimide-coated optical fiber and commercial optical backscattering reflectometry (OBR), which is a polarization-diverse implementation of OFDR that allows ultra-high spatial resolution. The team demonstrated a 20 m sensing distance with a dual-sensing fiber configuration for mutual decoupling and simultaneous measurement of temperature and RH. The humidity sensitivity is 0.281 GHz/%RH between 20%RH and 80%RH, and the response time is 20 min. A total of 5 cm spatial resolution was reported with a 20 m sensing distance. Ruishu F. Wright et al. [229] presented a different approach to distributed humidity sensing using a pre-loaded optical fiber under strain, where a section that is exposed to humidity or water changes its strain response and is thus detected using OBR. The reported response time is 60 min, and the dynamic range is 0%RH–100%RH.

### 3.5. Functional Materials

There have been numerous functional materials applied to optical fibers as a coating to serve as a transducer to the target measurand, as shown in Table 4. Generally, parameters such as sensitivity, specificity, response time, uniformity, robustness, toxicity, commercial availability, fabrication complexity, and cost are taken into account when choosing the right material. In recent years where innovations in optical techniques are limited, the focus has been on developing new materials to enhance the sensitivity, specificity, and/or response time. One particular area of interest is in-vivo-compatible materials that can be used for biomedical applications without hazardous side-effects, including internal monitoring via plugins and external monitoring via smart clothing.

## 4. Discussions

### 4.1. General Challenges

A large number of functional materials are composite and synthetic, which are either expensive or non-degradable. In addition, the production of sensitized composites is usually accompanied by by-products that cause environmental pollution [187]. It is necessary to find natural sensitization alternatives to realize biocompatibility such that deployed sensors are environmentally friendly. Biomaterials have natural degradability, biocompatibility, and environmental friendliness.

There are environments where water vapor is not the only component in addition to regular air, such as methane or carbon monoxide. To distinguish between a humid environment and one that is something else requires the functional material to be engineered in such a way that it only reacts to water vapor (i.e., high specificity). Otherwise, false readings will render the humidity sensor useless. As for miniaturization, the reduction in the dimensions of sensors allows for the less-intrusive deployment or installation in space-limited areas. The challenge is to downscale components while maintaining precision and robustness. The ability to readily integrate with electronic systems enables multiplexing as well as the adoption of the IOT, which will play an increasingly bigger role in smart cities of the future. Seamless integration relies on the use of electronic components within a reasonable package size to perform the necessary conversions and amplifications to match the impedance and connector of the rest of the system.

Cost reduction and lower maintenance add to the feasibility of employing such sensors and keeping them operational for long-term usage. One of the main limiters to the widespread use of specific sensors is the cost of upscaling and difficulty of maintenance. The hurdle to overcome includes mass production of the necessary components to lower the cost, as well as a simple design with long-lasting materials to minimize the need for regular maintenance. Protective membranes or components may be required to protect humidity sensors from dust and other forms of contamination, which will degrade the reliability of data over long.

In terms of tradeoffs, thin functional coatings or thin optical fibers tend to offer a faster response at the cost of a lower sensitivity [119]. Thick coatings or porous MMFs usually provide a higher sensitivity but slower response. The choice of coating parameters can be optimized based on the application requirements.

### 4.2. Specific Applications

There are challenges associated with particular applications, ranging from respiratory monitoring to nuclear power plants. This section discusses the specific needs and difficulties to overcome for achieving a viable sensing solution.

#### 4.2.1. Respiratory Monitoring

High levels of humidity can cause respiratory irritation [168], especially if the patient suffers from chronic lung diseases such as chronic obstructive pulmonary disease (COPD) or asthma. The optical response of the sensor is proportional to the humidity released from the mouth, also proportional to the distance between the sensor head and the mouth opening. After the moisture dries, problems arise if soluble substances, including salt or sugar, leave residue and accumulate near the sensor head. The materials of the sensor exposed to the measurement environment need to possess chemical durability, such as that of silicon and gold, which prevents rust and corrosion over a long service life.

#### 4.2.2. Portable Wearable Healthcare Products

Considering the wearable nature of the application, the humidity sensor construction materials need to be able to cope with frequent or constant large strains and bending effects [158]. Further, miniaturization for smart textiles or smart skin applications is needed to minimize intrusion. Relatively low-temperature cross-sensitivity is also very important to product design, as the human body emits varying levels of heat.

#### 4.2.3. Human Health Monitoring

Some clinical applications, such as laryngectomy, result in a bypass of the upper respiratory tract. As a result, the microclimate conditions of the nasal cavity and pharynx experience changes in the local temperature and RH [189]. This can lead to medical complications such as increased sputum volume and chronic lung disease, and ultimately abnormal nasal air conditions. Another example is the precise control of temperature and RH during mechanical ventilation of critically ill patients because there is a risk of respiratory mucosal damage caused by air-induced drying and cooling. In chronic wound applications, the rate of wound healing depends on temperature and RH. High RH contributes to impregnation, while low RH can lead to dry wounds. Hence, monitoring the RH of the wound micro-environment helps to gain an insight into the healing process.

#### 4.2.4. Grain Storage

Mildew is a critical problem in grain storage [180]. If unchecked, mildew can spread and cause huge losses in yield. Therefore, it is very important to monitor the conditions of mold breeding. It is necessary to monitor the RH distribution over a large area of stored grain, to help regulate ventilation and the extent of pesticide usage. By understanding the temperature and humidity parameters of a grain body, the changes in grain quality can be predicted. This informed planning mitigates the onset conditions of mycotoxins, particularly aflatoxin. Grain storage environments are typically harsh due to airborne dust particles from the grains. Dust particles can attach themselves to different parts of sensors, notably the sensor head. As a result, sensors used in grain piles require specially designed housing to minimize the effects of dust.

#### 4.2.5. Power Systems

The operational safety of transmission lines of power systems is subject to various environmental factors [194], including temperature, humidity, wind direction, wind speed, tower inclination. The transmission line operation and maintenance department monitor the real-time state of transmission lines to understand any anomalies in temperature and humidity distributions.

#### 4.2.6. Nuclear Power Plant

Nuclear power plants tend to employ condensate flow monitoring, sewage pool monitoring, and inspection of the main coolant inventory balance, allowing the total leakage to be measured quantitatively [176]. However, these methods are not sensitive enough or lack the means to detect local leakage along pipelines. Thermal imaging, ultrasonic and acoustic imaging methods are suitable for analyzing the defects of pipelines in nuclear power plants. This is because such methods are not distributed and thus require manual point-by-point inspection, which bears the risk of radiation exposure when radioactive coolant leaks in the main cooling system of the nuclear power plant. Therefore, remote real-time RH or chemical monitoring of coolant leakage using distributed fiber-optic sensors can ensure the safety of the operators.

## 5. Conclusions and Outlook

This review covered a wide range of humidity sensors reported in the literature to date. The motivation behind developing optical humidity sensors is clear, as humidity is an important parameter in a number of industries for quality control. The design choices and various challenges for different applications are also discussed. Fiber gratings, absorption loss, and micro-resonators lead the way in terms of design popularity. Some trends were observed between sensitivity, LOD, and response time, though not definitive due to the absence of a large set of reported parameters of interest. Some of the main difficulties in designing a practical humidity sensor for harsh environments include biocompatibility and contamination of the sensor head by the environment. The different functional materials used to sensitize the sensor heads are listed to offer an overview of the variety and to highlight the importance of material engineering in the field of sensors.

From the trajectory of existing developments, future advances in humidity sensing are anticipated to strive toward higher specificity, better miniaturization, more compatible system/environment integration, lower costs, and lower maintenance. Better limit of detection is meaningless without mass demand for such accuracy, and thus existing specifications can meet the majority of requirements in this area. New materials, particularly 1D and 2D structures and nanoscale-precision engineering techniques, will drive the research and development of novel humidity sensors that can adapt to the demand of industries and also pave the way for new applications. Reliability and reproducibility are likely to be better with the steadily improving automated manufacturing tools elevated by Industry 4.0.

## Figures and Tables

**Table 1:** Ranking of limit of detection.

Paper	Limit of Detection	Response Time	Sensitivity	Dynamic Range
2020, Anuj K. Sharma et al. [168]	0.0041%RH		171.11 dB/%RH for 99%RH	0%RH–100%RH
2021, Xin Cheng et al. [200]	0.0119%RH		84 MHz/%RH between 45%RH and 95%RH range	45%RH–95%RH
2019, Oskar Arrizabalaga et al. [155]	0.04%RH		148 pm/%RH between 10%RH and 95%RH	10%RH–95%RH
2019, George Y. Chen et al. [145]	0.099%RH or 0.007%RH	115 ms	0.04 dB/%RH between 10%RH–60%RH and 80%RH–94%RH, or 0.57 dB/%RH between 60%RH and 80%RH	10%RH–94%RH
2013, Jinesh Mathew et al. [86]	0.07%RH	75 ms	64 pm/%RH between 25%RH and 40%RH 137 pm/%RH between 40%RH and 90%RH	25%RH–90%RH
1991, W. Lukosz et al. [23]	0.1%RH	100 ms		
2012, W. Zhang et al. [81]	0.12%RH	12 min	33.6 pm/%RH between 30%RH and 90%RH	30%RH–90%RH
2012, Wei Chang Wong et al. [70]	0.5%RH, 0.14%RH or 0.04%RH	300 ms	0.04 nm/%RH, 0.15 nm/%RH, 0.60 nm/%RH at 50%RH, 70%RH, 90%RH	50%RH–80%RH
2017, Habibah Mohamed et al. [121]	0.177%RH		5.17 μW/% between 45%RH and 80%RH	45%RH–80%RH
2004, Alberto Alvarez-Herrero et al. [34]	0.2%RH	10 s	460 pm/%RH between 1%RH and 15%RH	1%RH–80%RH

**Table 2:** Ranking of response time.

Paper	Limit of Detection	Response Time	Sensitivity	Dynamic Range
2017, George Y. Chen et al. [118]	1.6%RH	3 ms	2.7%/%RH between 60%RH and 80%RH	0%RH–100%RH
2008, Fuxing Gu et al. [49]		30 ms		35%RH–88%RH
2012, Jinesh Mathew et al. [72]		50 ms	0.1 dB/%RH between 35%RH and 75%RH	25%RH–90%RH
2019, Miguel Hernaez et al. [153]		50 ms		20%RH–90%RH
2018, Cheng Li et al. [135]	3.1%RH 0.4%RH	60 ms	82 pm/%RH between 10%RH and 70%RH 630 pm/%RH between 70%RH and 90%RH	10%RH–90%RH
2008, Lei Zhang et al. [50]		70 ms		9%RH–94%RH
2017, Jia Shi et al. [124]	5%RH	72 ms	0.202 dB/%RH between 25%RH and 95%RH	25%RH–95%RH
2013, Jinesh Mathew et al. [87]	0.07%RH	75 ms	64 pm/%RH between 25%RH and 40%RH 137 pm/%RH between 40%RH and 90%RH	25%RH–90%RH
1991, W. Lukosz et al. [23]	0.1%RH	100 ms		
2009, Diana Viegas et al. [55]		100 ms		20%RH–80%RH

**Table 3:** Selection of electrical humidity sensors.

Reference	Response Time	Sensitivity
2014, U. Mogera et al. [204]	8 ms	~10,000
2013, Stefano Borini et al. [205]	30 ms	
2016, Rui Guo [206]	0.4 s	7.68 nF/%RH
2015, Anderson D. Smith et al. [207]	0.6 s	0.31($\frac{\Delta R}{R\cdot \Delta \%RH}$)
2014, Dongzhi Zhang et al. [208]	<1 s	1552.3 pF/%RH
2018, Xianhao Le et al. [209]	8 s	42.08 kHz/%RH
2013, Hengchang Bi et al. [210]	10.5 s	37800%($\frac{\Delta c}{c_{15}}\cdot 100\%$)
2018, Ishrat Rahim et al. [211]	10 s	154 nF/%RH
2019, Zhou Zheng et al. [212]	10 s	2.9 kHz (22.5–43.2%RH) 11.5 kHz (43.2–93.6%RH)
2017, Hui Yang et al. [213]	12 s	0.022($\Delta log_{10}^{c}∕\Delta RH$)
2012, Yao Yao et al. [214]	19 s	29 µV/%RH
2018, Tian Qiang et al. [215]	25 s	1.24 pF/%RH
2019, Ning Sun et al. [216]	27 s	177791.63%($\frac{\Delta C}{C_{11.3}}\cdot 100\%$)
2018, Debasree Burman et al. [217]	92 s	4000($\frac{I_{humidity}-I_{baseline}}{C_{baseline}}$)
2016, Jinfeng Feng et al. [218]		4.2($\frac{C}{C_{0}}$)

**Table 4:** Table of functional materials used for humidity sensing.

Material	Usage
Polyimide	[183,193,228]
Polyvinyl alcohol (PVA)	[54,115,162]
GO	[9,185,218]
Hydrogel	[150,169,185]
Agarose	[99,120,165]
Gelatin	[37,50]
Chitosan	[73,167,188]
Poly(diallyldimethylammonium) (PDDA)/poly R-478	[222]
Poly(allylamine hydrochloride) (PAH) and poly(acrylic acid)(PAA)	[92]
Hydroxyethylcellulose (HEC)/polyvinylidenefluoride (PVDF)	[91]
Zinc oxide (ZnO)	[129,140,216]
Tin(IV) oxide (SnO_2_)	[93,184]
Titanium dioxide (TiO_2_)	[77]
Methylene blue/hydroxypropyl cellulose	[149]
Photonic cellulose nanocrystals	[16]
Poly(vinyl alcohol-co-vinyl acetal)s	[17]
Silica gel	[59,78]
Silk fibroin	[160]
Gold nanoparticles linked by polyethylene glycol	[198]
Poly methyl meth acrylate (PMMA)	[162,211,223]
Molybdenum disulfide (MoS2) or tungsten disulfide (WS2)-protected titanium	[164,190]
Calcium alginate (CaAlg) hydrogel	[169]
Niobium disulfide	[169]
Sodium silica	[82]
Poly(3,4-ethylenedioxythiophene) polystyrene sulfonate in PSS (PEDOT:PSS) in polyvinyl alcohol (PVA)	[67]
Polyvinylidene fluoride in dimethyl sulfoxide (DMSO) and hydroxyethyl cellulose (HEC)	[147]
Acrylic resin	[177]
Carboxymethyl cellulose (CMC)/carbon nanotubes (CNTs)	[178]
nickel-doped zinc thin oxide (NZTO) perovskite	[56]
Polystyrene (PS) in poly(N-isopropylacrylamide) (PNIPAM)	[185]
Spider dragline silk	[186]
Polydimethylsiloxane (PDMS)	[187]
Poly(allylamine hydrochloride) (PAH)/silica (SiO_2_) nanoparticles	[17]
Graphene quantum dots	[197]
